# Genetic disorder prenatal diagnosis and pregnancy termination practices among high consanguinity population, Saudi Arabia

**DOI:** 10.1038/s41598-019-53655-8

**Published:** 2019-11-21

**Authors:** Sayed AbdulAzeez, Nourah H. Al Qahtani, Noor B. Almandil, Amani M. Al-Amodi, Sumayh A. Aldakeel, Neda Z. Ghanem, Deem N. Alkuroud, Ameen AlTurki, Quds Abdulhakeem AlQattan, Abdulrahman Alghamdi, Norah Fahad Alhur, Hatoon Ahmed Al Taifi, Halah Egal Aljofi, B. Rabindran Jermy, Vinoth Raman, Antonino Giambona, Aurelio Maggio, J. Francis Borgio

**Affiliations:** 1 Department of Genetic Research, Institute for Research and Medical Consultation (IRMC), Imam Abdulrahman Bin Faisal University, Dammam, 31441 Saudi Arabia; 2 Department of Obstetrics and Gynaecology, College of Medicine, Imam Abdulrahman Bin Faisal University, Dammam, 31441 Saudi Arabia; 3 Department of Clinical Pharmacy Research, Institute for Research and Medical Consultation (IRMC), Imam Abdulrahman Bin Faisal University, Dammam, 31441 Saudi Arabia; 4 Department of Environmental Health Research, Institute for Research and Medical Consultation (IRMC), Imam Abdulrahman Bin Faisal University, Dammam, 31441 Saudi Arabia; 5 Department of Nano Medicine Research, Institute for Research and Medical Consultation (IRMC), Imam Abdulrahman Bin Faisal University, Dammam, 31441 Saudi Arabia; 6 Deanship of Quality and Academic Accreditation, Imam Abdulrahman Bin Faisal University, Dammam, 31441 Saudi Arabia; 7 Unit of Hematology for Rare Diseases of the Blood and Blood-forming Organs, Laboratory for Molecular Diagnosis of Rare Diseases, Hospital Villa Sofia Cervello, Palermo, 90145 Italy; 8 Campus of Haematology Franco and Piera Cutino, AOOR Villa Sofia-V. Cervello, Palermo, 90146 Italy

**Keywords:** Consanguinity, Genetics research

## Abstract

The prevalence of consanguineous marriage and genetic disorders are high in Saudi Arabia. There were records on the practices of Saudis toward prenatal diagnosis (PND) and termination of pregnancy (TOP), however the sample sizes are small. This study has targeted the Saudi Arabian community and family history of genetic disorders to determine the practices toward PND and TOP. The cross-sectional survey was conducted among Saudis (*n* = 2761) to determine their practices toward reproductive-decision making. Regression analysis was conducted to identify the association of the limiting factors, relative merits and family history on the outcomes. Total of 2507 participants returned completed questionnaire. The practice towards PND (68%) were more favorable than TOP (33%). PND was found to be a good opportunity for early diagnosis and gives parent’s choice. Education, history with affected baby, prior knowledge and religious belief were significant deciding factors of PND and TOP. Down syndrome (*n* = 161) and sickle cell anemia (*n* = 152) were commonly available genetic disorder among participant’s family. Respondents with autistic cases in their family have higher acceptance rate for TOP. Non-consanguineous are more willing to consider TOP than consanguineous. Participants with abnormal fetus, aged of > 36 years, married and educated Saudis were more likely consider TOP. Though, religion is the most influencing factor for not accepting TOP, comparatively willingness to PND and TOP have increased recently. Awareness campaigns about PND and TOP may increase the chances of accepting prenatal genetic diagnosis.

## Introduction

Scientific advancements in genetic testing have moved us to an era of individualized preventive medicine. Genetic testing has the maximum possible potential to reduce the prevalence of genetic disorders by early detection and making strategized decisions for prevention^1^.Studies found that general knowledge of genetics in Arab countries lacks understanding the fundamental characteristics of genetic diseases^2^. This is consequently a major concern in Eastern countries, which have high prevalence of inherited genetic diseases, primarily due to the high occurrence of consanguineous marriages^3,4^. Consanguinity or blood relative marriage is union between close biological relatives from the same kinship as another person. Saudi Arabia is one of the top four countries with the highest prevalence (42.1–66.7%) of consanguineous marriages as the majority of the marriages are still tribal^3^. This lead to a substantial burden of genetic diseases in the country^4^.

For the purpose of eliminating such burden of genetic diseases, patients are required to make decisions concerning genetic testing. The understanding and the practice of patients toward genetic testing would mainly influence their medical decisions^5^. For this reason, several studies have been conducted in Saudi Arabia in an effort to investigate the attitudes of specific groups in the community towards Prenatal Diagnosis (PND) and Termination of Pregnancy (TOP) for certain or hypothetical genetic disease(s) that the fetus could have^6^. Although the studies were done on small sample size in which the largest sample used was 400 Saudi parents^6^, these studies have revealed a majority with favorable attitude toward PND; however, TOP was less accepted among participants. Religious belief has been shown to be the main influence on the participants’ practices concerning PND and TOP^7–9^.

Although many studies have investigated the attitudes of Saudis toward PND and TOP, they focused on either specific groups of the community^2,8^ or a particular condition^7,8^ with relatively small sample size and were region specific. This study, we are targeting the cross section of the Saudi Arabian community; males and females, both married and single with wide-range of ages and educational backgrounds as well as covering a diverse number of genetic diseases. In Saudi Arabia where the prevalence of consanguineous marriage is still high, we found it is mandatory to conduct such a study, considering that the current premarital screening methods are not fulfilling the objectives of screening^10,11^. In particular, the main objective of the study is to determine the practices toward PND and TOP

## Methods

This cross-sectional survey was conducted among Saudi adults from January 25^th^ to April 3^rd^, 2018 in different regions (Eastern Region, Central Region, Hijaz Region, Northern Region, and Southern Region) of Saudi Arabia. The questionnaires were sent by online (email or WhatsApp) or handed out to Saudi males and females, both married and single. The questionnaire was designed in accordance with previously published literature and included additional questions in accordance with the local circumstances (Fig. S1)^2,11^. It was pre-tested for validation among healthcare providers and modified based on the Institutional Review Boards (IRB) committee guidelines^12^. All methods were performed in accordance with the relevant guidelines and regulations of the National Committee of Bioethics (NCBE), Saudi Arabia. Informed consent (singed consent on handed out; consent paragraph was included in the beginning of the Google Forms in place of a separate consent form and participants were requested register their willingness to participate) was obtained from all participants. The survey was conducted in both English and Arabic languages depending on the respondent’s preference. The survey instrument took approximately 5 minutes to be completed.

The questionnaire comprised two sections (Fig. S1); the first section focused on socioeconomic and background information such as gender, age, education level, marital status, previous experience with unhealthy baby pregnancy and abortion. While the second section asked about the knowledge of prenatal diagnosis, PND advantages and disadvantages, family history of inherited diseases and the outcomes on whether participants would consider prenatal diagnosis and their practice toward termination of pregnancy if fetus was diagnosed with a genetic disease. Outcome variables of the study are TOP and PND, their response were ‘yes’, ‘no’, ‘depends on severity’ and ‘not sure’ (Table S1).

The International Classification of Diseases-10 (ICD-10) was used to classify (14 groups) the family history of genetic disorders^13^ (Table S2). The study also conducts a qualitative analysis, which explains the views of the participants on the advantages and disadvantages over the prenatal genetic screening (Table S3). Advantages pointed by the participants were categorized into 1. Early diagnosis and correction of genetic abnormalities; 2. Awareness and medical planning; 3. The choice of abortion; and 4. Psychological readiness. While the disadvantages expressed by the respondents were categorized into six categories (Psychological pressure, Invasive procedure, Inaccurate diagnosis, Against faith, Expensive, and The choice of abortion). The choice of abortion was common between advantages and disadvantages depending on the participants’ view. Some answers have been translated from Arabic to English (Table S3).

Respondents who did not consent to participate in the study, below the age of 18 and/or did not answer the questions of the study outcome i.e. practice towards prenatal diagnosis and termination of affected embryos were excluded from the study. Ethical approval was obtained from the Institutional Review Boards Committee of the Imam Abdulrahman Bin Faisal University (IRB-2017-13-137).

Data analyzed using the IBM (International Business Machines Corporation) SPSS (Statistical Package for the Social Sciences) Statistics version 23 (IBM Co., Armonk, New York, United States of America). Continuous data were described as descriptive statistics (means and standard deviations), while categorical data were presented as frequencies and percentages. At bivariate level, Chi-square test was used to investigate which of the variables were associated significantly to practices toward both PND and TOP. Multivariate logistic regression analyses were carried out to predict which explanatory variables (age, marital status, education and reason) were associated significantly with practices toward PND and TOP. All variables that showed association with practices at the bivariate level with *p*-value less than 0.05 and < 0.01 were considered statistically significant and highly significant respectively and were entered into the regression analyses. The confidence level was set at 95% and 99%. The missing values were excluded from the outcome and explanatory variables, which reduce the sample size but not influenced the regression analysis (Table S1).

## Results

### Sample characteristics

**Figure 1:**
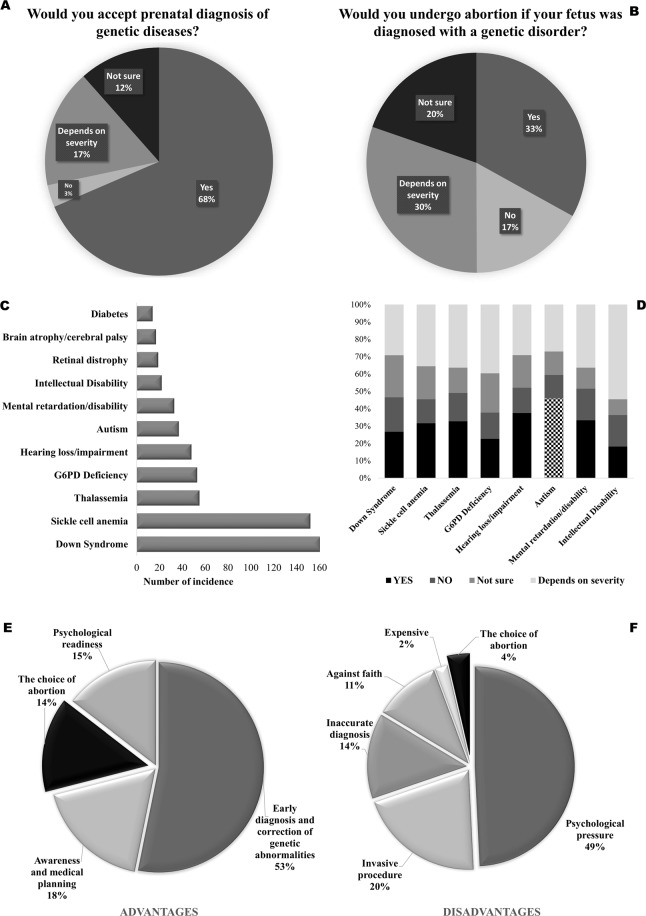
Overall practices towards Prenatal Diagnosis (PND) and Termination of Pregnancy (TOP) in Saudi participants with/without genetic disorders history. (**A**) Overall practice towards PND of genetic disorders; (**B**) Overall practice towards TOP in case of an affected fetus with a genetic disorder; (**C**) Number of incidence of genetic disorders history reported in the survey; (**D**) Practice towards TOP among participants with history of most prevalent genetic disorders; (**E**) The advantages and (**F**) Disadvantages addressed by the respondents regarding prenatal diagnosis.

In total, 2761 (handed out 44 + 2717 by online) individuals responded to the questionnaire, of whom 2507 (90.8%; Margin of error 1.957% at 95% confidence) submitted a completed questionnaire and 39 (1.41%) have not met the inclusion criterion. In response to the questionnaires sent by handed out, 25.14% (7♂, 36♀) of the questionnaires were returned. Table 1 displays the characteristics of the study sample. Majority of the respondents were female (overall 82.7%; handed out 81.81%; online 82.73%), > 36 years old (63.0%), married (79.0%), and almost half of the respondents (57.1%) were undergraduates. Thirty-four percent of the diseases were in the ‘blood and blood-forming organs and certain disorders involving the immune mechanism’ (BD) category, 25.2% ‘Congenital malformation, deformations and chromosomal abnormalities’ category, and 13.6% Mental and behavior disorders category (Table S2). Although BD represents the highest percentage as a disease sub-group, Down syndrome (*n* = 161) was the most commonly reported disorder followed by sickle cell anemia (*n* = 152) (Fig. 1).

**Table 1:** Characteristics & background information of the respondents.

Variables	Frequency (*n* = 2761*) *n* (%)
**Marital Status**	
Yes	2181 (79.0)
No	513 (18.6)
**Married to a relative**	
Yes	830 (30.1)
No	1719 (62.3)
**Been pregnant with an affected baby**	
Yes	260 (9.4)
No	2145 (77.7)
**Undergone abortion before**	
Yes	957 (34.7)
No	1450 (52.5)
**Ever diagnosed your fetus for genetic diseases before delivery**	
Yes	85 (3.1)
No	2309 (83.6)
**Family history of genetic diseases**	
Yes	741 (26.8)
No	2016 (73.0)
**Gender**	
Male	318 (11.5)
Female	2284 (82.7)
**Age**	
18–25	405 (14.7)
26–30	456 (16.5)
31–35	59 (2.1)
36 and above	1740 (63.0)
**Education**	
High School	644 (23.3)
Undergraduate	1577 (57.1)
Post-graduate	507 (18.4)
No education	14 (0.5)
**Heard of ‘prenatal diagnosis’**	
Yes	1374 (49.8)
No	1343 (48.6)

*Total respondents without excluding any missing values.

### Practices toward prenatal diagnosis

The majority (68%) would consider prenatal diagnosis in a future pregnancy (Fig. 1). The total responses for PND advantages and disadvantages were *n* = 948, and *n* = 198 respectively. All the advantages and disadvantages over the PND were categorized based on the opinion of the respondents (Table S3). Most of the responses related to the advantages were regarding early diagnosis and correction of genetic abnormalities (*n* = 504, 53%) as for disadvantages, psychological pressure (*n* = 100, 49%) was the highest (Fig. 1).

The association between sociodemographic characteristics and practice toward PND at the bivariate level is displayed in Table 2. The practice was significantly associated with level of education (*p* < 0.0001), age (*p* = 0.005), previous pregnancy with an affected baby (*p* = 0.039) and having a prior knowledge of prenatal diagnosis (*p* = 0.001). Nevertheless, having a family history of inherited diseases did not significantly alter the respondents’ practice to prenatal diagnosis (*p* = 0.461).

**Table 2:** Bivariate association of practices toward prenatal diagnosis (PND) and termination of pregnancy (TOP) with different characteristics of the respondents.

Variables	13.Practice toward PND			14.Practice toward TOP		
	*Mean*	*SD*	*p-value*^a^	*Mean*	*SD*	*p-value*^a^
**Marital Status**						
Yes	1.69	1.097	0.508	2.35	1.150	**0**.**021**
No	1.76	1.147		2.41	1.097	
**Married to a relative**						
Yes	1.65	1.073	0.192	2.40	1.137	**0**.**031**
No	1.72	1.110		2.33	1.157	
Never Married^b^	1.76	1.149		2.41	1.096	
**Been pregnant with an affected baby**						
Yes	1.60	1.018	**0**.**039**	2.08	1.095	**9**.69E-06*
No	1.72	1.113		2.38	1.152	
Never Married^b^	1.72	1.132		2.46	1.070	
**Undergone abortion before**						
Yes	1.71	1.111	0.350	2.36	1.144	**0**.**001**
No	1.70	1.099		2.34	1.153	
Never Married^b^	1.72	1.133		2.46	1.071	
**Ever diagnosed your fetus for genetic diseases before delivery**						
Yes	1.49	0.994	0.355	2.09	1.116	**0**.**000237**
No	1.71	1.107		2.36	1.149	
Never Married^b^	1.72	1.133		2.46	1.071	
**Family history of genetic diseases**						
Yes	1.69	1.115	0.416	2.36	1.130	0.562
No	1.71	1.104		2.37	1.144	
**Gender**						
Male	1.61	1.078	0.06	2.35	1.170	0.256
Female	1.72	1.110		2.37	1.136	
**Age**						
18–25	1.60	1.040	**0**.**005**	2.34	1.115	**0**.**001**
26–30	1.66	1.083		2.33	1.207	
31–35	1.81	1.093		2.18	1.088	
36 and above	1.74	1.127		2.39	1.129	
**Education**						
High School	1.81	1.167	**1**.48E-06*	2.45	1.098	**3**.1509E-05*
Undergraduate	1.73	1.111		2.35	1.137	
Post-graduate	1.52	0.996		2.30	1.192	
No education	1.57	0.938		2.00	1.240	
**Heard of ‘prenatal diagnosis’**						
Yes	1.63	1.063	0.**001**	2.33	1.142	0.193
No	1.79	1.145		2.39	1.138	
**Reason for TOP answer**^**c**^						
Religion				2.63	1.052	**1**.27E-91*
Ethics				2.44	1.097	
Culture				1.75	0.754	
Other				1.83	1.140	

SD: Standard Deviation; ^a^p value of the chi-square test. Bold values are statistically significant at 0.05 level; ^b^Represent the group who said No to marital status; PND: prenatal diagnosis; TOP: Termination of pregnancy; ^**c**^The reason was requested only of the TOP.

### Practices toward termination of pregnancy

Figure 1 gives the relative frequencies with which the different practices were held. Seventeen percent of respondents felt that TOP is ‘unacceptable’, whereas 33% felt that it is ‘acceptable’ and 30% felt it is acceptable under certain circumstances. Acceptability of TOP varied between respondents with a family history. Those with more autism in the family history (45%) reporting a favorable practice than individuals with Down syndrome in their family history (25%) (Fig. 1).

Bivariate analysis revealed a statistical difference in favor for TOP for the following variables; marital status (*p* = 0.021), married to a relative (*p* = 0.031), been pregnant with an affected baby (*p* = 9.69 × 10^−6^), undergone abortion before (*p* = 0.001), ever diagnosed fetus for genetic diseases (*p* = 0.0002), age (*p* = 0.001) and education (*p* = 3.15 × 10^−5^). Practices were comparable between gender, who had a family history of genetic diseases, and prior knowledge of prenatal diagnosis. The religious belief is one of the most influencing and highly significant (*p* = 1.12 × 10^−91^) factor affecting TOP consideration (Table 2). Multivariate logistic regression analysis (unadjusted and adjusted) disclosed that people between the age of 31–35 (OR = 0.379, 95% Cl = 0.176–0.817; *p* = 0.013) years compared with people > 36 years were less likely to go for TOP than not willing to go TOP (Table 3).

**Table 3:** Multivariate Logistic regression analysis of termination of pregnancy (TOP) on other variables.

Variables		Unadjusted			Adjusted		
		Odds ratio	95% CI	*p-value*	Odds ratio	95% CI	*p-value*
Age	31–35	0.379	0.176–0.817	0.013*	0.379	0.172–0.836	0.016*
	26–30	1.137	0.789–1.638	0.491	1.005	0.698–1.445	0.961
	18–25	0.879	0.599–1.289	0.509	1.174	0.817–1.687	0.386
Married		1.674	1.188–2.358	0.003*	0.935	0.680–1.285	0.680
Education	Postgraduate	0.282	0.064–1.238	0.093	3.048	0.301–30.876	0.346
	Undergraduate	0.362	0.084–1.570	0.175	3.102	0.309–31.190	0.336
	High School	0.807	0.181–3.591	0.778	5.459	0.533–55.890	0.153
Reason	Others	0.655	0.182–2.358	0.517	3.902	0.745–20.440	0.107
	Ethics	2.502	0.665–9.413	0.175	10.970	2.026–59.405	0.005*
	Religion	8.385	2.283–30.788	0.001*	8.018	1.496–42.983	0.015*

*significant at 95% confidence interval.

## Discussion

The current study examined the practice of the cross section of the Saudi Arabian community regarding PND and TOP for genetic disorders, in addition to investigating the factors that contribute to their practices. Conducting such a study is highly needed especially in Saudi Arabia where genetic disorders are relatively common due to the high prevalence of consanguineous marriages. In addition to the consanguineous marriages, the high maternal and paternal ages and the tendency to have large families as well as lack of health measures contributes to the increase of prevalence of such genetic diseases^4^. Eliminating such burden of genetic disorders depends, at least partially, on public knowledge and practice toward genetic testing^5^.

Favorable practices toward PND practice was significantly influenced by higher education levels, older age, prior pregnancy with an affected baby and having a prior knowledge of prenatal diagnosis. Our findings are in line with other studies reporting fairly favorable practices toward PND^7^. However, despite the positive practices toward PND, participants held a more critical practice when it comes to TOP. Many factors contribute to the acceptance of TOP in addition to be educated (undergraduate and higher education levels), young in age, and prior pregnancy with an affected baby. People who are married and married to non-relative are more willing for TOP. Furthermore, people who had abortion before and had a fetus diagnosed with genetic diseases were more in favor of TOP.

Although the majority of participants felt that TOP is ‘acceptable’, it is worth noting that their acceptance was conditioned by performing Islamic regulations of abortion. Whereas, the main reason for not accepting abortion was religion. Religion seems to be a major factor in participants’ practice toward TOP as previously reported in other studies^7^. The Islamic regions believe that ensoulment occurs after 120 days of pregnancy and as a result, TOP would be forbidden as the fetus is considered a living human^14^. Before 120 days of pregnancy, TOP can be done if the fetal genetic or non-genetic condition is incompatible with life after birth or there will be a great disability and suffering. It is permitted after 120 days when continuing the pregnancy would risk the mother’s life^14^. The Council of senior scientists in the Kingdom provided the Ministry of Health (MOH) with an advisory opinion (fatwah) regarding abortion as the following: “Abortion should not be done without a medical decision from a specialized committee that can be trusted, and that committee has to have at least 3 Muslim doctors, or if there is no Muslim doctor then it is ok. Also, abortion cannot be done without the approval of both parents or the mother alone if there is direct harm to her only”.

Here we observed a high acceptance toward TOP in individuals with autistic cases in their family (Fig. 1), unlike previous studies that showed a favorable practice toward TOP in hypothetical Down syndrome case compared to autism^9,15^. Both genetic diseases reported to cause a stress in the family in coping with the conditions^16^, however, they do perceive the severity of the genetic diseases differently^9^. Severity of the genetic diseases has a major impact on the acceptance of TOP among families with a history of intellectual disability (Fig. 1). In comparison to the previous study, there is a marginal decrease toward PND and TOP. The previous study focused on a well-educated region and only college students. To add, the study did not refine abortion as a single option^2^. Contradictorily, the married respondents or their wives ever been pregnant with an affected baby were more likely to prefer TOP, while they were less likely to consider PND. This might be due to the previous experience of having psychological pressure followed by the diagnosis of an affected baby. There was a marginal increase on the rate acceptance toward PND by the respondents, those who addressed the advantages of PND. One individuals’ response towards the benefits or advantages towards prenatal diagnosis of genetic diseases was “prenatal diagnosis helps in protecting the lives of the child as well as the whole family from the disease burden and the negative aspects of the society”. Furthermore, majority of the responses addressed the advantages of PND. This clearly reflects the positive thinking of the studied population. However, we cannot neglect the view held by a minority of people (0.97%) with the impact of the inaccurate PND and against faith. Moreover, only little percentage of participants felt that the PND is disadvantageous due to psychological pressure or the invasive nature of the procedure.

In 2016 (25^th^ April), the King and Crown Prince of Saudi Arabia announced the Saudi Vision 2030, which is a strategic plan to improve all aspects of life in the Kingdom^17^. The Ministry of Health (MOH) is part of the National Transformation Programs in the vision and one of its main objectives is to improve the quality of healthcare services using prevention and therapeutic approaches to control diseases (National Transformation Program 2020). Within the upcoming years the Kingdom is going to undergo tremendous changes to achieve a modernized healthcare system by implementing several programs that seek to educate the public by raising awareness on important health issues^18^. The present report on the survey would collectively change the publics’ knowledge and practice toward PND and TOP^15^.

The study has various strengths, this is the first of its kind of cross-sectional survey with a large number of respondents, the addition of parameters about the positive and negative effects of PND have been included for the first time along with reproductive-decision making. The comparative analysis on reproductive-decision making with additional influencing factors like various disease histories is worth mentioning. The study has several limitations; It is not mandatory that the observation of the study population would translate into real decision on PND and TOP^19^. The study missed the opportunity to look at practices to different types of prenatal diagnosis (invasive/non-invasive). We believe that our results reflect the practices the Saudis, but final decisions are always subjected to change based on family situation. The study did not include any couple. Major limitation of the study is most of the data were collected through online sources.

## Conclusion

Early diagnosis is a good opportunity for the respondents and it gives parent’s choice. Respondents with no history of an affected baby were more likely to prefer PND. Education, prior knowledge of PND and history of affected baby are significant decisive factors for PND; while education, history of affected baby, abortion and religious beliefs are the most influencing decisive factors for TOP. The acceptance rate of PND is nearly double than TOP. Respondents with autism in their family history were more accepting TOP. Saudis who are married to non-relative are more willing for TOP than those in a consanguineous marriage. Saudis with a history of a fetus diagnosed with genetic diseases, aged > 36 years and married respondents or their wives ever been pregnant with an affected baby were more likely prefer TOP. Despite religion being a meritorious factor on deciding on a TOP, practice towards PND and TOP have been moderately positive among the respondents. Many parents want prenatal testing to obtain information that will allow them to prepare for life with a child who has a genetic condition. However, more awareness and detailed education about PND and TOP may increase the chances of accepting genetic diagnosis and planning positive strategies in the future.

## Supplementary information

Figure S1, Table S1. Table S2 and Table S3
