# Supplementary material for: Genetic disorder prenatal diagnosis and pregnancy termination practices among high consanguinity population, Saudi Arabia
Source: Sci Rep. 2019 Nov 21;9:17248. doi: 10.1038/s41598-019-53655-8 (PMC6872573; doi:10.1038/s41598-019-53655-8)
Supplement: Supplementary file 1 — Figure S1, Table S1. Table S2 and Table S3 [file 41598_2019_53655_MOESM1_ESM.pdf]

Genetic disorder prenatal diagnosis and pregnancy termination practices among high consanguinity population, Saudi Arabia

Sayed AbdulAzeez[1], Nourah H Al Qahtani[2], Noor B Almandil[3], Amani M. Al-Amodi[1], Sumayh A. Aldakeel[1], Neda Z. Ghanem[1], Deem N. Alkuroud[1], Ameen AlTurki[1], Quds Abdulhakeem AlQattan[1], Abdulrahman Alghamdi[1], Norah Fahad Alhur[1], Hatoon Ahmed Al Taifi[2], Halah Egal Aljofi[4], B. Rabindran Jermy[5], Vinoth Raman[6], Antonino Giambona[7], Aurelio Maggio[8], J. Francis Borgio[1]

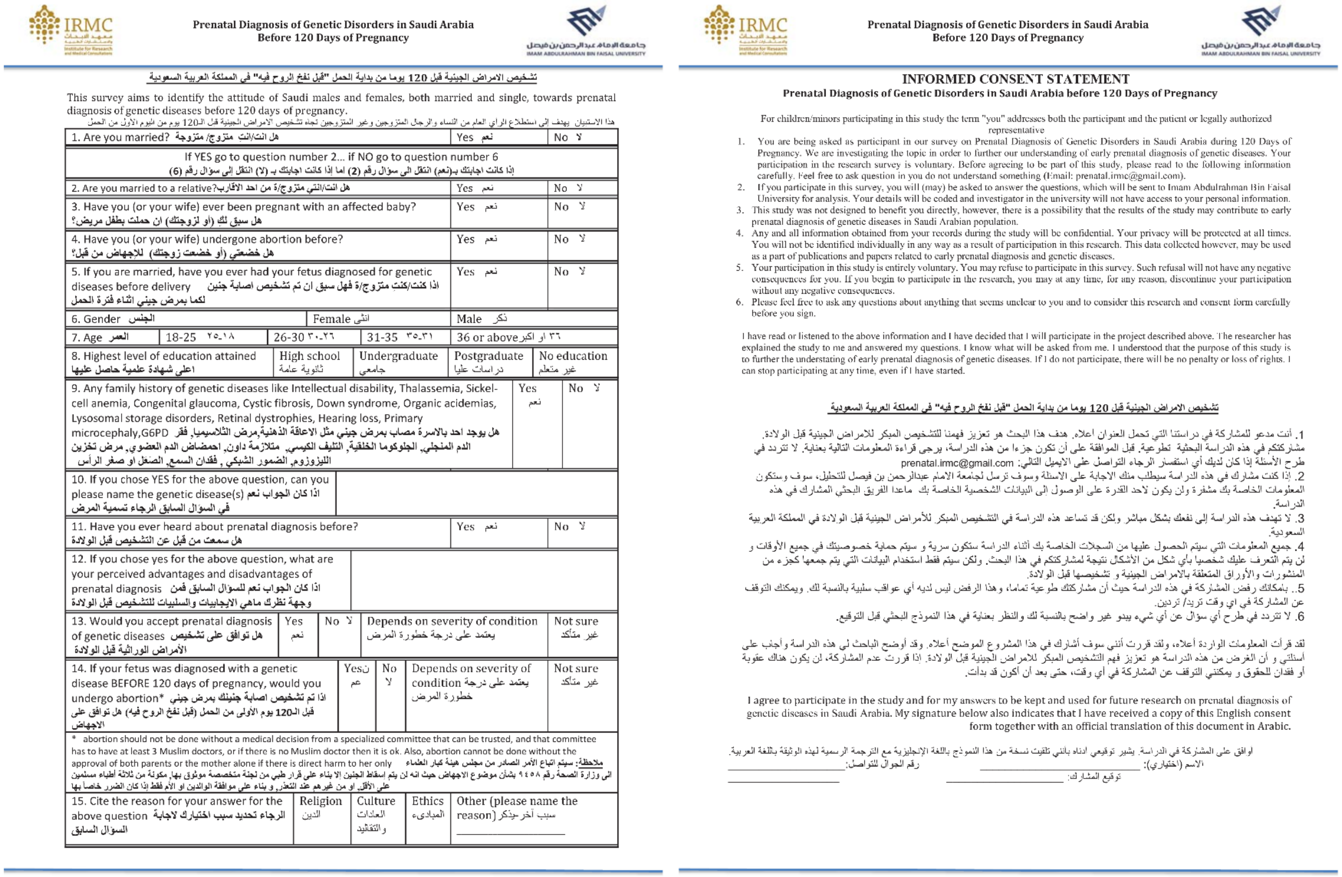

Prenatal Diagnosis of Genetic Disorders in Saudi Arabia
Before 120 Days of Pregnancy

تشخيص الامراض الجينية قبل 120 يوما من بداية الحمل "قبل نفخ الروح فيه" في المملكة العربية السعودية

This survey aims to identify the attitude of Saudi males and females, both married and single, towards prenatal diagnosis of genetic diseases before 120 days of pregnancy.

هذا الاستبيان يهدف إلى استطلاع الرأي العام من النساء والرجال المتزوجين وغير المتزوجين تجاه تشخيص الامراض الجينية قبل الـ120 يوم من اليوم الأول من الحمل

1. Are you married? هل انت/انتِ متزوج/ متزوجة			Yes نعم	No لا
If YES go to question number 2... if NO go to question number 6<br>إذا كانت اجابتك بـ(نعم) انتقل الى سؤال رقم (2) اما إذا كانت اجابتك بـ (لا) انتقل إلى سؤال رقم (6)				
2. Are you married to a relative? هل انت/انتي متزوج/ة من احد الاقارب			Yes نعم	No لا
3. Have you (or your wife) ever been pregnant with an affected baby? هل سبق لكِ (أو لزوجتك) ان حملت بطفل مريض؟			Yes نعم	No لا
4. Have you (or your wife) undergone abortion before? هل خضعتي (أو خضعت زوجتك) للإجهاض من قبل؟			Yes نعم	No لا
5. If you are married, have you ever had your fetus diagnosed for genetic diseases before delivery اذا كنت/كنتِ متزوج/ة فهل سبق ان تم تشخيص اصابة جنين لكما بمرض جيني اثناء فترة الحمل			Yes نعم	No لا
6. Gender الجنس		Female انثى	Male ذكر	
7. Age العمر	18-25 ١٨-٢٥	26-30 ٢٦-٣٠	31-35 ٣١-٣٥	36 or above ٣٦ او اكبر
8. Highest level of education attained اعلى شهادة علمية حاصل عليها	High school ثانوية عامة	Undergraduate جامعي	Postgraduate دراسات عليا	No education غير متعلم
9. Any family history of genetic diseases like Intellectual disability, Thalassemia, Sickle-cell anemia, Congenital glaucoma, Cystic fibrosis, Down syndrome, Organic acidemias, Lysosomal storage disorders, Retinal dystrophies, Hearing loss, Primary microcephaly,G6PD هل يوجد احد بالاسرة مصاب بمرض جيني مثل الاعاقة الذهنية,مرض الثلاسيميا, فقر الدم المنجلي, الجلوكوما الخلقية, التليف الكيسي, متلازمة داون, احمضاض الدم العضوي, مرض تخزين الليزوزوم, الضمور الشبكي , فقدان السمع, الصَعَل او صغر الرأس			Yes نعم	No لا
10. If you chose YES for the above question, can you please name the genetic disease(s) اذا كان الجواب نعم في السؤال السابق الرجاء تسمية المرض				
11. Have you ever heard about prenatal diagnosis before? هل سمعت من قبل عن التشخيص قبل الولادة			Yes نعم	No لا
12. If you chose yes for the above question, what are your perceived advantages and disadvantages of prenatal diagnosis اذا كان الجواب نعم للسؤال السابق فمن وجهة نظرك ماهي الايجابيات والسلبيات للتشخيص قبل الولادة				
13. Would you accept prenatal diagnosis of genetic diseases هل توافق على تشخيص الأمراض الوراثية قبل الولادة	Yes نعم	No لا	Depends on severity of condition يعتمد على درجة خطورة المرض	Not sure غير متأكد
14. If your fetus was diagnosed with a genetic disease BEFORE 120 days of pregnancy, would you undergo abortion* اذا تم تشخيص اصابة جنينك بمرض جيني قبل الـ120 يوم الأولى من الحمل (قبل نفخ الروح فيه) هل توافق على الاجهاض	Yesن عم	No لا	Depends on severity of condition يعتمد على درجة خطورة المرض	Not sure غير متأكد
* abortion should not be done without a medical decision from a specialized committee that can be trusted, and that committee has to have at least 3 Muslim doctors, or if there is no Muslim doctor then it is ok. Also, abortion cannot be done without the approval of both parents or the mother alone if there is direct harm to her only ملاحظة: سيتم اتباع الأمر الصادر من مجلس هيئة كبار العلماء الى وزارة الصحة رقم ٩٤٥٨ بشأن موضوع الاجهاض حيث انه لن يتم إسقاط الجنين إلا بناء على قرار طبي من لجنة متخصصة موثوق بها, مكونة من ثلاثة أطباء مسلمين على الأقل, او من غيرهم عند التعذر, و بناء على موافقة الوالدين او الأم فقط إذا كان الضرر خاصاً بها				
15. Cite the reason for your answer for the above question الرجاء تحديد سبب اختيارك لاجابة السؤال السابق	Religion الدين	Culture العادات والتقاليد	Ethics المبادىء	Other (please name the reason) سبب آخر-يذكر ________

Prenatal Diagnosis of Genetic Disorders in Saudi Arabia
Before 120 Days of Pregnancy

INFORMED CONSENT STATEMENT

Prenatal Diagnosis of Genetic Disorders in Saudi Arabia before 120 Days of Pregnancy

For children/minors participating in this study the term "you" addresses both the participant and the patient or legally authorized representative

1. You are being asked as participant in our survey on Prenatal Diagnosis of Genetic Disorders in Saudi Arabia during 120 Days of Pregnancy. We are investigating the topic in order to further our understanding of early prenatal diagnosis of genetic diseases. Your participation in the research survey is voluntary. Before agreeing to be part of this study, please read to the following information carefully. Feel free to ask question in you do not understand something (Email: prenatal.irmc@gmail.com).
2. If you participate in this survey, you will (may) be asked to answer the questions, which will be sent to Imam Abdulrahman Bin Faisal University for analysis. Your details will be coded and investigator in the university will not have access to your personal information.
3. This study was not designed to benefit you directly, however, there is a possibility that the results of the study may contribute to early prenatal diagnosis of genetic diseases in Saudi Arabian population.
4. Any and all information obtained from your records during the study will be confidential. Your privacy will be protected at all times. You will not be identified individually in any way as a result of participation in this research. This data collected however, may be used as a part of publications and papers related to early prenatal diagnosis and genetic diseases.
5. Your participation in this study is entirely voluntary. You may refuse to participate in this survey. Such refusal will not have any negative consequences for you. If you begin to participate in the research, you may at any time, for any reason, discontinue your participation without any negative consequences.
6. Please feel free to ask any questions about anything that seems unclear to you and to consider this research and consent form carefully before you sign.

I have read or listened to the above information and I have decided that I will participate in the project described above. The researcher has explained the study to me and answered my questions. I know what will be asked from me. I understood that the purpose of this study is to further the understating of early prenatal diagnosis of genetic diseases. If I do not participate, there will be no penalty or loss of rights. I can stop participating at any time, even if I have started.

تشخيص الامراض الجينية قبل 120 يوما من بداية الحمل "قبل نفخ الروح فيه" في المملكة العربية السعودية

1. أنت مدعو للمشاركة في دراستنا التي تحمل العنوان اعلاه. هدف هذا البحث هو تعزيز فهمنا للتشخيص المبكر للامراض الجينية قبل الولادة. مشاركتكم في هذه الدراسة البحثية تطوعية. قبل الموافقة على أن تكون جزءا من هذه الدراسة، يرجى قراءة المعلومات التالية بعناية. لا تتردد في طرح الأسئلة إذا كان لديك أي استفسار الرجاء التواصل على الايميل التالي: prenatal.irmc@gmail.com
2. إذا كنت مشارك في هذه الدراسة سيطلب منك الاجابة على الاسئلة وسوف ترسل لجامعة الامام عبدالرحمن بن فيصل للتحليل، سوف وستكون المعلومات الخاصة بك مشفرة ولن يكون لاحد القدرة على الوصول إلى البيانات الشخصية الخاصة بك ماعدا الفريق البحثي المشارك في هذه الدراسة.
3. لا تهدف هذه الدراسة إلى نفعك بشكل مباشر ولكن قد تساعد هذه الدراسة في التشخيص المبكر للأمراض الجينية قبل الولادة في المملكة العربية السعودية.
4. جميع المعلومات التي سيتم الحصول عليها من السجلات الخاصة بك أثناء الدراسة ستكون سرية و سيتم حماية خصوصيتك في جميع الأوقات و لن يتم التعرف عليك شخصيا بأي شكل من الأشكال نتيجة لمشاركتكم في هذا البحث. ولكن سيتم فقط استخدام البيانات التي يتم جمعها كجزء من المنشورات والأوراق المتعلقة بالامراض الجينية و تشخيصها قبل الولادة.
5. . بامكانك رفض المشاركة في هذه الدراسة حيث أن مشاركتك طوعية تماما، وهذا الرفض ليس لديه أي عواقب سلبية بالنسبة لك. ويمكنك التوقف عن المشاركة في اي وقت تريد/ تردين.
6. لا تتردد في طرح أي سؤال عن أي شيء يبدو غير واضح بالنسبة لك والنظر بعناية في هذا النموذج البحثي قبل التوقيع.

لقد قرأت المعلومات الواردة أعلاه، ولقد قررت أنني سوف أشارك في هذا المشروع الموضح أعلاه. وقد أوضح الباحث لي هذه الدراسة وأجاب على أسئلتي و أن الغرض من هذه الدراسة هو تعزيز فهم التشخيص المبكر للامراض الجينية قبل الولادة. إذا قررت عدم المشاركة، لن يكون هناك عقوبة أو فقدان للحقوق و يمكنني التوقف عن المشاركة في أي وقت، حتى بعد أن أكون قد بدأت.

I agree to participate in the study and for my answers to be kept and used for future research on prenatal diagnosis of genetic diseases in Saudi Arabia. My signature below also indicates that I have received a copy of this English consent form together with an official translation of this document in Arabic.

اوافق على المشاركة في الدراسة. يشير توقيعي ادناه بأنني تلقيت نسخة من هذا النموذج باللغة الإنجليزية مع الترجمة الرسمية لهذه الوثيقة باللغة العربية.

الاسم (اختياري): ________ رقم الجوال للتواصل: ________

توقيع المشارك: ________ ________

Figure S1. The bilingual questionnaire used in the study.

Table S1. List of categories used for the analysis before and after curation.

Question in the survey	Code	Response	No. of responses before curation (n = 2761)	No. of responses after curation (n =2507)
**1. Are you married?**	1	Yes	2181	2034
	2	No	513	473
		Blank	67	-
**2. Are you married to a relative**	1	Yes	830	751
	2	No	1719	1290
	3	Blank/ Never married	212	466
**3. Have you (or your wife) ever been pregnant with an affected baby?**	1	Yes	260	250
	2	No	2145	1950
	3	Blank/ Never married	356	307
**4. Have you (or your wife) undergone abortion before?**	1	Yes	957	855
	2	No	1450	1346
	3	Blank/ Never married	354	306
**5. If you are married, have you ever had your fetus diagnosed for genetic diseases before delivery**	1	Yes	85	80
	2	No	2309	2121
	3	Blank/ Never married	367	306
**6. Gender**	1	Male	318	304
	2	Female	2284	2203
		Blank	159	-
**7. Age**	1	31- 35	456	57
	2	26 - 30	405	436
	3	18 - 25	350	386
	4	36 and above	1449	1628

		Blank	101	-
**8. Highest level of education attained**	1	High school	644	573
	2	Undergraduate	1577	1445
	3	Post-graduate	507	475
	4	No education	14	14
		Blank	19	-
**9. Any family history of genetic diseases**	1	Yes	744	700
	2	No	2016	1807
		Blank	1	-
**11. Have you ever heard about prenatal diagnosis before**	1	1.Yes	1399	1275
	2	2.No	1361	1232
		Blank	1	
**13.Would you accept prenatal diagnosis of genetic diseases**	1	Yes	1889	1725
	2	No	87	75
	3	Depends on severity	463	423
	4	Not sure	318	284
		Blank	4	-
**14. If your fetus was diagnosed with a genetic disorder BEFORE 120 days of pregnancy, would you undergo abortion**	1	Yes	912	841
	2	No	466	412
	3	Depends on severity	838	756
	4	Not sure	544	498
		Blank	1	-
**15. Cite the reason for your answer for the above question**	1	Religion	1571	1404
	2	Ethics	364	342
	3	Other	670	749
	4	Culture	14	12
		Blank	142	-

Table S2. List of genetic disorders and diseases among the Saudi Arabians and their prevalence in Saudi Arabia; classification of diseases based on the ICD-10 (International Statistical Classification of Diseases and Related Health Problems 10th Revision).

Classification (Total number of response)	Disease	Number of responses	Prevalence in Saudi Arabia	Reported / Prevalence in KSA
Diseases of Nervous System (34)	Brain atrophy/Cerebral palsy	17	0.41 %	Al-Rajeh et al., 1991/ Al-Asmari et al., 2006
	Chorea disease (Huntington's Chorea)	1	3-4 per 100,000	Bohlega et al., 1995/ Scrimgeour, 2009
	Demyelinating neuropathy * (Demyelination neuropathy)	1	-	Bolino, et al., 2000
	Multiple sclerosis	1	25 per 100,000	Yaqub and Daif, 1988$
	Epilepsy	7	6.5 per 1000	Al-Rajeh et al., 1990/ Khan, 2015
	Charcot–Marie–Tooth disease	1	-	Al-Owain et al., 2012
	Dystonia disease	1	-	Malibary et al., 2004
	Quadriplegia	1	-	Al-Owain et al., 2012
	Hydrocephalus	2	1.6 per 1000	Alkuraya, 2014<br>Al-Dosari et al., 2013/ Murshid et al., 2000
	Paralysis*	1	-	-
	Parkinson's Disease	1	0.27 per 1000	Al Rajeh et al., 1993$
Congenital malformation, deformations and chromosomal	Cockayne syndrome	2	-	Mahmoud et al., 2002
	klippel feil syndrome	1	-	Alkuraya, 2014<br>Mohamed, et al., 2013
	Microcephaly	4		Alkuraya, 2014/ Faheem et al, 2015

abnormalities (195)	Tuberous sclerosis	1	14 to 17 per 100,000	Seyam et al., 2008/ Al-Orainey et al., 2013
	Macrocephaly*	1	Neural tube defects in KSA is 1.2 per 1000	Al-Mendalawi, 2015[$]
	Anencephaly	1	0.43 per 1000	Asindi, et al., 2001/ El-Awad and Sivasankaran, 1992
	Down Syndrome	161	1.8 per 1000	Al Husain, M., 2003/ Niazi et al., 1995
	Angelman's syndrome	2	0.2 per 100,000	Al-Salloum et al., 2015[$]
	Fragile x	3	0.4 to 0.8 in males and 0.2 to 0.6 per 1000 in females	Iqbal et al., 2000[$]
	Prader–Willi syndrome	1	-	Al Sarkhy et al., 2014
	spina bifida defect	2	1.09 per 1000	Murshid, 2000[$]
	Meyer syndrome	1	-	#
	Epidermolysis bullosa	1	Unknown	ABAHUSSEIN et al., 1993/ Alhumidi, 2015
	Ichthyosis vulgaris	2	7 per 1000	Alkuraya, 2014<br>Aldahmesh et al., 2011/ Al-Zayir, 2006
	Adult polycystic kidney disease	1	-	Hussein et al., 1994.
	Carpenter's syndrome	1	-	Twigg, et al., 2012
	Prune belly syndrome	2	-	Abdurrahman, and Elidrissy, 1988.
	Sanjad-Sakati syndrome	1	-	Al-Owain et al., 2012<br>Parvari et al., 2002

	Turner syndrome	1	0.2 per 10000	Al Alwan et al., 2014/ Al-Salloum et al., 2015
	Cutis laxa	1	-	Allanson et al., 1986
	Congenital glaucoma	5	1 per 2500	Bejjani et al., 1998/ Badeeb, 2011
Endocrine, nutritional and metabolic diseases (42)	Diabetes	14	23.9 %	Alqurashi et al., 2011/ Naeem, 2015
	Acute intermittent porphyria	1	-	Alfadhel et al., 2016.
	Galactosemia	1	12 per 100,000	Al Aqeel, 2004/ Moammar et al., 1996
	Acidosis*	3	-	-
	Hemostatic metabolism of urea (homocystinuria)	1	-	Al-Owain et al., 2012 Zaidi, et al., 2012
	Hurler's Syndrome	1	-	Al Aqeel, 2004
	Lysosomal storage disease	2	42.2 per 100,000	Alkuraya, 2014/ Al-Sannaa et al., 2017
	Niemann-Pick disease	1	1 per 100,000	Al Aqeel, 2004/ Moammar et al., 2010
	Tyrosinemia	1	3 out of 100,000	Imtiaz et al., 2011 Al-Essa et al.,1998/ Moammar et al., 2010
	Cholesterol*	2	Dyslipidemi ranges from 20% to 40% in KSA	Alkaabba et al., 2012[$]
	Citrullinemia	1	4 in 100,000	Alsadah and Ansari, 2017/ Moammar et al., 2010
	Adrenal Insufficiency (Addison's disease)	1	-	Al Kandari et al., 2016
	Thyroid diseases*	1	43.6 %	Albasri, et al., 2014/ Ali and Altahir, 2016

	Adrenal gland atrophy *	1	-	
	Hypothyroidism	1	40.8 %	Alsadah and Ansari, 2017/ Ali and Altahir, 2016
	Albinism	1	-	Harfi, et al., 1992
	Cystic Fibrosis	7	1 in 4243	Al-Owain et al., 2012/ Banjar, et al., 1999
	Slight rise in blood lactate (Lactic acidosis)*		150 per 100,000 (this is not specific for lactic acidosis, this is for inborn errors of metabolism)	Moammaret al., 2010$
	Amino acids in the blood (Aminoacidopathy)		150 per 100,000 (this is not specific foraminoacidopathy, this is for inborn errors of metabolism)	Al Aqeel, 2004/ Moammaret al., 2010
Diseases of the blood and blood-forming organs and certain disorders involving the immune mechanism (265)	Anemia/Sickle cell anemia		2.6 %	Alkuraya, 2014/ Jastaniah, 2011
	G6PD Deficiency	53	4.76 %	El-Hazmi and Warsy, 1988/ Alharbi and Khan, 2014
	Thalassemia	55	3.4 % for beta thalassemia trait	Alkuraya, 2014/ Al-Suliman, 2006
	Glanzmann's thrombasthenia	1	-	Ahmed et al., 1988$
	Hemophilia C	2	Overall prevalence of 5.3% for Hepatitis C not Haemophilia C in Saudi Arabia	Bahakim, et al., 1991$
	Blood diseases*	1	13.6 per 1000 for B thalassemia and 49.6 for SCD out of 1000	Al Sulaiman, et al., 2008/ Alsaeed et al., 2017
	Hereditary blood disease* blood diseases*	1	13.6 per 1000 for B thalassemia and 49.6 for SCD out of 1000	Al Sulaiman, et al., 2008/ Alsaeed et al., 2017
Mental and	Neurasthenia	1	-	#

behavior disorders (105)	Autism	37	1.4 to 29 per 10,000	Al-Salehi et al., 2009/ Salhia et al., 2014
	Intellectual Disability/delayed learning	22	8.9 per 1000	Alkuraya, 2014/ El Hazmi et al., 2003
	Attention deficit disorder with Hyperactivity	2	1.3 to 16% hyperactive ADHD and 1.4 to 7.8% inattention type	Al-Modayfer and Alatiq, 2015/ Alhraiwil et al., 2015
	Mild-Severe Mental retardation/disability	33	8.9 per 1000	Alkuraya, 2014/ El Hazmi et al., 2003
	Bipolar disorder	5	Minor mental illness morbidity prevalence 18.2 %	Al-Khathami and Ogbeide, 2002[$]
	Schizophrenia	1	Minor mental illness morbidity prevalence 18.2 %	Al-Khathami and Ogbeide, 2002[$]
	Depression	2	35.7 %	Al-Khathami and Ogbeide, 2002/ Becker et al., 2002
	Psychiatric illness	2	Minor mental illness morbidity prevalence 18.2 %	Al-Khathami and Ogbeide, 2002[$]
Certain infectious and parasitic diseases (5)	Hepatitis C	1	Overall prevalence of 5.3% of Hepatitis C not Haemophilia C in Saudi Arabia	Abdelaal, et al., 1994/ Bahakim, et al., 1991
	Poliomyelitis	1	-	-
	Meningitis	1	40 per 100,000	Almuneef et al., 1998/ Almuneef et al., 2001
Diseases of the genitourinary system (3)	Renal failure	1	5.7 %	Al Homrany, 2003<br>Kari, J.A., 2006/ Alsuwaida et al., 2010
	Hypertrophy of kidney*	1	11% (out of 95 patients) hypertensive nephroscelorsis	Alkhunaizi, A.M., 2007[$]

	Stone in kidney	1	111 per 100,000	Ramello et al., 2001/ Alkhunaizi, 2016
Diseases of the digestive system (2)	Coeliac disease/ wheat allergy	2	3.1 % Qassim<br>2.1% Aseer<br>1.8% Madinah	Al Attas, 2002/ Aljebreen et al., 2013
Diseases of the ear and mastoid process (48)	Hearing loss/impairment	47	1.75 %	Alkuraya, 2014/ Al-Rowaily 2012
	Deaf-mutism	1	Less than 1% (selective mutism)	Hassan et al., 2013[$]
Diseases of the eye and adnexa (17)	Retinal dystrophy	10	2.9% Retinal Disease	Alkuraya, 2014/ Darraj et al., 2016
	Nearsightedness	2	21.3 %	Al Wadaani et al., 2013/ Darraj et al., 2016
	ophthalmic diseases*	1	-	
	vision impairment	2	13.9 %	Al-Rajhi et al., 1993/ Al-Shaaln et al., 2011
	Retinal pigement (retinitis pigmentosa)	1	-	Aldahmesh et al., 2009
	Distorting the eye mesh*	1	-	-
Diseases of the musculoskeletal system and connective tissue (16)	Bethlem myopathy	1	-	-
	Muscle atrophy*	11	3.1 % LGMD2L	Bohlega et al., 2015[$]
	Clubfoot	3	-	Jawadi, 2010
	Scleroderma	1	-	Rajapakse, 1987
Diseases of the circulatory system (7)	Narrow P artery (stenosis)	1	6.6 % (carotid stenosis)	Ahmed et al., 2015
	High blood pressure (Hypertension)	1	26.1 %	Al-Nozha et al., 2007[$]

	Coronary/heart	5	5.5 %	Al-Nozha et al., 2004[$]
Diseases of the skin and subcutaneous tissue (4)	Grey hair*	1	-	
	Baldness*	3	6.75 % males and 6.375 % females (these results are for alopecia)	AM, A.E.M. and Maghrabi, 2015
Neoplasms (4)	Colon cancer	1	9.6 per 100,000	Mansoor et al., 2002/ Alsanea et al., 2015
	Breast cancer	1	19.2 per 100,000	Ezzat et al., 1999/ Saggu et al., 2015
	Cancer*	1	78.1 per 100,000 for males while for females 86.7 per 100,000	Taylor, J.W., 1963/ Bazarbashi et al., 2017
	Leukemia	1	5.2 per 100,000 (Riyadh) 4.9 per 100,000 both Eastern and Northern Region while in Najran 4.5 per 100,000	El-Siss et al., 2006/ Alghamdi et al., 2014
Responses with symp* (30)	Immediate death after birth	1	Neonatal mortality rate 21.4 per 1000 while infantile mortality rate 53.8 per 1000 and post neonatal death rate was 32.5 per 1000	Al-Nahedh, 1997[$]
	The birth of a mutilated embryo	1	-	
	Antibodies	1	-	
	Atrophy	1	-	
	Inert tissue in the left lung	1	-	
	Lack of oxygen	3	-	
	Liver	1	-	

	Hole in the head	1	-	
	Constitutionally *	1	-	
	Diasability /loss of movement/Physical disability	5	1.7%	Al-Jadid, 2014$
	Tortuosity syndrome	1	-	Faiyaz-Ul-Haque et al., 2009
	Change in chromosome 23	1	-	
	Blood acidity (Organic acidemias)	1	1 in 2000 to 1 in 5000 depending on region	Alkuraya, 2014/ Al Essa et al. (1998)
	Comprehensive developmental delay gene KLCM5	1	-	-
	Delayed growth	2	11 %	El Mouzan et al., 2012$
	Simple weakness in memory	2	-	-
	Shortness of memory	1	-	-
	Biliray atresia	1	The incidence of EHBA is unknown	Meng et al., 1997/ Crankson and Abdullah, 2001
	DART	1	-	-
	Confusion/ lack of concentration	2	36.1 %	Basheikh et al., 2017$
	Mutism	1	Less than 1%	Hassan et al., 2013$
	Ascites	2	-	Khan, et al., 2008

No reference available
*Description by the respondents are insufficient
The description in the parenthesis describe the disease from the respondent’s answer
$ The reference is for both reporting and prevalence of the disorder in KSA

Bibliography

Abahussein, A.A., Al-Zayir, A.A., Mostafa, W.Z. And Okoro, A.N., 1993. Epidermolysis bullosa in the eastern province of Saudi Arabia. *International journal of dermatology*, *32*(8), pp.579-581.

Abdelaal, M., Rowbottom, D., Zawawi, T., Scott, T. and Gilpin, C., 1994. Epidemiology of hepatitis C virus: a study of male blood donors in Saudi Arabia. *Transfusion*, *34*(2), pp.135-137.

Abdurrahman, M.B. and Elidrissy, A.T., 1988. Childhood renal disorders in Saudi Arabia. Pediatric Nephrology, 2(3), pp.368-372.

Ahmed, A., Ahmad, M. and Van Onselen, R., 2015. 9. Prevalence and impact of carotid disease in adult Saudi patients undergoing isolated coronary artery bypass surgery on early post-operative outcome. *Journal of the Saudi Heart Association*, *27*(4), p.302.

Ahmed, M.A., Al-Sohaibani, M.O., Al-Mohaya, S.A., Sumer, T., Al-Sheikh, E.H. and Knox-Macaulay, H.K., 1988. Inherited bleeding disorders in the Eastern Province of Saudi Arabia. Acta haematologica, 79(4), pp.202-206.

Aida Al Aqeel, M.D., 2004. Common genetics and metabolic diseases in Saudi Arabia. Middle East Journal of Family Medicine, 6(6).

Al Alwan, I., Khadora, M., Amir, I., Nasrat, G., Omair, A., Brown, L., Al Dubayee, M. and Badri, M., 2014. Turner Syndrome Genotype and phenotype and their effect on presenting features and timing of Diagnosis. International journal of health sciences, 8(2), p.195.

Al Attas, R.A., 2002. How common is celiac disease in Eastern Saudi Arabia. Ann Saudi Med, 22(5), pp.315-19.

Al Homrany, M., 2003. Epidemiology of acute renal failure in hospitalized patients: experience from southern Saudi Arabia.

Al Husain, M., 2003. Growth charts for children with Down's syndrome in Saudi Arabia: birth to 5 years. *International journal of clinical practice*, *57*(3), pp.170-174.

Al Kandari, H., Katsumata, N., Alexander, S. and Rasoul, M.A., 2006. Homozygous mutation of P450 side-chain cleavage enzyme gene (CYP11A1) in 46, XY patient with adrenal insufficiency, complete sex reversal, and agenesis of corpus callosum. *The Journal of Clinical Endocrinology & Metabolism*, *91*(8), pp.2821-2826.

Al Rajeh, S., Bademosi, O., Ismail, H., Awada, A., Dawodu, A., Al-Freihi, H., Assuhaimi, S., Borollosi, M. and Al-Shammasi, S., 1993. A community survey of neurological disorders in Saudi Arabia: the Thugbah study. *Neuroepidemiology*, *12*(3), pp.164-178.

Al Sarkhy, A., Alasmi, M., Assiri, A.M. and ab Fowzan, S.A., 2014. Congenital hepatic fibrosis in a child with Prader-Willi syndrome: a novel association. Annals of Saudi medicine, 34(1), p.81.

Al Sulaiman, A., Suliman, A., Al Mishari, M., Al Sawadi, A. and Owaidah, T.M., 2008. Knowledge and attitude toward the hemoglobinopathies premarital screening program in Saudi Arabia: population-based survey. *Hemoglobin*, *32*(6), pp.531-538.

Al Wadaani, F.A., Amin, T.T., Ali, A. and Khan, A.R., 2013. Prevalence and pattern of refractive errors among primary school children in Al Hassa, Saudi Arabia. Global journal of health science, 5(1), p.125.

Al-Asmari, A., Al Moutaery, K., Akhdar, F., & Al Jadid, M. (2006). Cerebral palsy: incidence and clinical features in Saudi Arabia. Disability and rehabilitation, 28(22), 1373-1377.

Albasri, A., Hussainy, A.S., Alhujaily, A. and Sawaf, Z., 2014. Histopathological patterns of thyroid disease in Al-Madinah region of Saudi Arabia

Aldahmesh, M.A., Mohamed, J.Y., Alkuraya, H.S., Verma, I.C., Puri, R.D., Alaiya, A.A., Rizzo, W.B. and Alkuraya, F.S., 2011. Recessive mutations in ELOVL4 cause ichthyosis, intellectual disability, and spastic quadriplegia. *The American Journal of Human Genetics*, *89*(6), pp.745-750.

Aldahmesh, M.A., Safieh, L.A., Alkuraya, H., Al-Rajhi, A., Shamseldin, H., Hashem, M., Alzahrani, F., Khan, A.O., Alqahtani, F., Rahbeeni, Z. and Alowain, M., 2009. Molecular characterization of retinitis pigmentosa in Saudi Arabia. Molecular vision, 15, p.2464.

Al-Dosari, M.S., Al-Owain, M., Tulbah, M., Kurdi, W., Adly, N., Al-Hemidan, A., Masoodi, T.A., Albash, B. and Alkuraya, F.S., 2013. Mutation in MPDZ causes severe congenital hydrocephalus. *Journal of medical genetics*, *50*(1), pp.54-58.

Al-Essa, M., Rashed, M. and Ozand, P.T., 1998. Tyrosinemia type II: Report of the first four cases in Saudi Arabia. Annals of Saudi medicine, 18(5), pp.466-468.

Alfadhel, M., Benmeakel, M., Hossain, M.A., Al Mutairi, F., Al Othaim, A., Alfares, A.A., Al Balwi, M., Alzaben, A. and Eyaid, W., 2016. Thirteen year retrospective review of the spectrum of inborn errors of metabolism presenting in a tertiary center in Saudi Arabia. Orphanet journal of rare diseases, 11(1), p.126.

Alghamdi, I.G., Hussain, I.I., Alghamdi, M.S., Dohal, A.A. and El-Sheemy, M.A., 2014. The incidence of leukemia in Saudi Arabia. Descriptive epidemiological analysis of data from the Saudi Cancer Registry (2001-2008). *Saudi Medical Journal*, *35*(7), pp.674-683.

Alharbi, K.K. and Khan, I.A., 2014. Prevalence of glucose-6-phosphate dehydrogenase deficiency and the role of the A− variant in a Saudi population. *Journal of International Medical Research*, *42*(5), pp.1161-1167.

Alhraiwil, N.J., Ali, A., Househ, M.S., Al-Shehri, A.M. and El-Metwally, A.A., 2015. Systematic review of the epidemiology of attention deficit hyperactivity disorder in Arab countries. *Neurosciences*, *20*(2), p.137.

Alhumidi, A.A., 2015. Hereditary Epidermolysis Bullosa in Saudi Arabia, Epidemiological, Pathological, Ultrastructural Study of Fourteen Patients.

Ali, A.A.G. and Altahir, S.A., 2016. Prevalence of Thyroids Dysfunction among Saudi Adult Males and Females from (June–September 2016).

Al-Jadid, M.S., 2014. Disability trends in Saudi Arabia: Prevalence and causes. *American journal of physical medicine & rehabilitation*, *93*(1), pp.S47-S49.

Aljebreen, A.M., Almadi, M.A., Alhammad, A. and Al Faleh, F.Z., 2013. Seroprevalence of celiac disease among healthy adolescents in Saudi Arabia. *World journal of gastroenterology: WJG*, *19*(15), p.2374.

Al-Kaabba, A.F., Al-Hamdan, N.A., El Tahir, A., Abdalla, A.M., Saeed, A.A. and Hamza, M.A., 2012. Prevalence and correlates of dyslipidemia among adults in Saudi Arabia: results from a national survey. *Open Journal of Endocrine and Metabolic Diseases*, *2*(04), p.89.

Al-Khathami, A.D. and Ogbeide, D.O., 2002. Prevalence of mental illness among Saudi adult primary-care patients in Central Saudi Arabia. Saudi medical journal, 23(6), pp.721-724.

Alkhunaizi, A.M., 2007. Pattern of renal pathology among renal biopsy specimens in Eastern Saudi Arabia. *Saudi medical journal*, *28*(11), pp.1676-1681.

Alkhunaizi, A.M., 2016. Urinary stones in Eastern Saudi Arabia. *Urology annals*, *8*(1), p.6.

Alkuraya, F.S., 2014. Genetics and genomic medicine in Saudi Arabia. Molecular genetics & genomic medicine, 2(5), pp.369-378.

Allanson, J., Austin, W. and Hecht, F., 1986. Congenital cutis laxa with retardation of growth and motor development: a recessive disorder of connective tissue with male lethality. Clinical genetics, 29(2), pp.133-136.

Al-Mendalawi, M.D., 2015. Epidemiology of neural tube defects. *Saudi Medical Journal*, *36*(3), p.373.

Al-Modayfer, O. and Alatiq, Y., 2015. A Pilot Study on the Prevalence of Psychiatric Disorders among Saudi Children and Adolescents: a Sample from a Selected Community in Riyadh City. Arab Journal of Psychiatry, 26(2), pp.184-192.

Almuneef, M., Alshaalan, M., Memish, Z. and Alalola, S., 2001. Bacterial meningitis in Saudi Arabia: the impact of Haemophilus influenzae type b vaccination. *Journal of chemotherapy*, *13*(sup1), pp.34-39.

Almuneef, M., Memish, Z., Khan, Y., Kagallwala, A. and Alshaalan, M., 1998. Childhood bacterial meningitis in Saudi Arabia. *Journal of Infection*, *36*(2), pp.157-160.

Al-Nahedh, N., 1997. Infant mortality in the rural Riyadh region of Saudi Arabia. *Journal of the Royal Society of Health*, *117*(2), pp.106-109.

Al-Nozha, M.M., Abdullah, M., Arafah, M.R., Khalil, M.Z., Khan, N.B., Al-Mazrou, Y.Y., Al-Maatouq, M.A., Al-Marzouki, K., Al-Khadra, A., Nouh, M.S. and Al-Harthi, S.S., 2007. Hypertension in Saudi Arabia. Saudi medical journal, 28(1), pp.77-84.

Al-Nozha, M.M., Arafah, M.R., Al-Mazrou, Y.Y., Al-Maatouq, M.A., Khan, N.B., Khalil, M.Z., Al-Khadra, A.H., Al-Marzouki, K., Abdullah, M.A., Al-Harthi, S.S. and Al-Shahid, M.S., 2004. Coronary artery disease in Saudi Arabia. Saudi medical journal, 25(9), pp.1165-1171.

Al-Orainey, I., Alhedaithy, M.A., Alanazi, A.R., Barry, M.A. and Almajid, F.M., 2013. Tuberculosis incidence trends in Saudi Arabia over 20 years: 1991-2010. *Annals of thoracic medicine*, *8*(3), p.148.

Al-Owain, M., Al-Zaidan, H. and Al-Hassnan, Z., 2012. Map of autosomal recessive genetic disorders in Saudi Arabia: concepts and future directions. *American Journal of Medical Genetics Part A*, *158*(10), pp.2629-2640.

Alqurashi, K.A., Aljabri, K.S. and Bokhari, S.A., 2011. Prevalence of diabetes mellitus in a Saudi community. Annals of Saudi medicine, 31(1), p.19.

Al-Rajeh, S., Abomelha, A., Awada, A., Bademosi, O. and Ismail, H., 1990. Epilepsy and other convulsive disorders in Saudi Arabia: a prospective study of 1,000 consecutive cases. Acta neurologica scandinavica, 82(5), pp.341-345.

Al-Rajeh, S., Bademosi, O., Awada, A., Ismail, H., Al-Shammasi, S. and Dawodu, A., 1991. CEREBRAL PALSY IN SAUDI ARABIA: A CASE-CONTROL STUDY OF RISK FACTORS. Developmental Medicine & Child Neurology, 33(12), pp.1048-1052.

Al-Rajhi, A.A., Al-Omar, O.M., Al-Ghamdi, S.A. and Jabak, M., 1993. Prevalence and causes of visual impairment and blindness in the south western region of Saudi Arabia. International ophthalmology, 17(3), pp.161-165.

Al-Rowaily, M.A., AlFayez, A.I., AlJomiey, M.S., AlBadr, A.M. and Abolfotouh, M.A., 2012. Hearing impairments among Saudi preschool children. *International journal of pediatric otorhinolaryngology*, *76*(11), pp.1674-1677.

Alsadah, Z. and Ansari, N., 2017. The frequency of inherited metabolic and endocrine disorders in the eastern and north-western Jawf provinces of Saudi Arabia: Four years data from the newborn screening department, Ministry of Health, Dammam. Current Pediatric Research, 21(4).

Alsaeed, E.S., Farhat, G.N., Assiri, A.M., Memish, Z., Ahmed, E.M., Saeedi, M.Y., Al-Dossary, M.F. and Bashawri, H., 2017. Distribution of hemoglobinopathy disorders in Saudi Arabia based on data from the premarital screening and genetic counseling program, 2011–2015. *Journal of Epidemiology and Global Health*.

Al-Salehi, S.M., Al-Hifthy, E.H. and Ghaziuddin, M., 2009. Autism in Saudi Arabia: presentation, clinical correlates and comorbidity. Transcultural Psychiatry, 46(2), pp.340-347.

AlSalloum, A., ElMouzan, M.I., AlHerbish, A., AlOmer, A. and Qurashib, M., 2015. Prevalence of selected congenital anomalies in Saudi children: a community-based study. *Annals of Saudi medicine*, *35*(2).

Alsanea, N., Abduljabbar, A.S., Alhomoud, S., Ashari, L.H., Hibbert, D. and Bazarbashic, S., 2015. Colorectal cancer in Saudi Arabia: incidence, survival, demographics and implications for national policies. *Annals of Saudi medicine*, *35*(3), p.196.

Al-Sannaa, N.A., Al-Abdulwahed, H.Y. and Al-Ghamdi, M.S., 2017. Lysosomal Storage Disorders (LSDs): The Prevalence in the Eastern Province of Saudi Arabia.

Al-Shaaln, F.F., Bakrman, M.A., Ibrahim, A.M. and Aljoudi, A.S., 2011. Prevalence and causes of visual impairment among Saudi adults attending primary health care centers in northern Saudi Arabia. *Annals of Saudi medicine*, *31*(5), p.473.

Al-Suliman, A., 2006. Prevalence of B-thalassemia trait in premarital screening in Al-Hassa, Saudi Arabia. *Annals of Saudi medicine*, *26*(1), p.14.

Alsuwaida, A.O., Farag, Y.M., Al Sayyari, A.A., Mousa, D., Alhejaili, F., Al-Harbi, A., Housawi, A., Mittal, B.V. and Singh, A.K., 2010. Epidemiology of chronic kidney disease in the Kingdom of Saudi Arabia (SEEK-Saudi investigators)-a pilot study. *Saudi Journal of Kidney Diseases and Transplantation*, *21*(6), p.1066.

Al-Zayir, A.A. and Al-Amro Al-Alakloby, O.M., 2006. Clinico-epidemiological features of primary hereditary ichthyoses in the Eastern province of Saudi Arabia. *International journal of dermatology*, *45*(3), pp.257-264.

AM, A.E.M. and Maghrabi, I.A., 2015. Prevalence and treatment of Alopecia areata in Taif area, KSA. *Prevalence*, *4*(2), pp.125-128.

Asindi, A. and Al-Shehri, A., 2001. Neural tube defects in the Asir region of Saudi Arabia. *Annals of Saudi medicine*, *21*(1/2), pp.26-29.

Badeeb, O.B., 2011. Congenital Glaucoma in Saudi Arabia. *King Abdulaziz University press, Jeddah*.

Bahakim, H., Bakir, T.M.F., Arif, M. and Ramia, S., 1991. Hepatitis C virus antibodies in high-risk Saudi groups. *Vox sanguinis*, *60*, pp.162-164.

Banjar, H., Kambouris, M., Meyer, B.F., Al-Mehaidib, A. and Mogarri, I., 1999. Geographic distribution of cystic fibrosis transmembrane regulator gene mutations in Saudi Arabia. *Annals of tropical paediatrics*, *19*(1), pp.69-73.

Basheikh, M.A., Alaama, T.Y., Sherbini, N.A., Alghamdi, W.S., Ahmad, A., 2017. THE PREVALENCE STUDY OF DELIRIUM IN ELDERLY HOSPITALIZED PATIENTS IN MEDICAL WARDS IN KING ABDULAZIZ UNIVERSITY HOSPITAL, JEDDAH: DETECTION, POSSIBLE CAUSES AND OUTCOMES. *Indian Journal of research*, *6*(10), pp.59-63.

Bazarbashi, S., Al Eid, H. and Minguet, J., 2017. Cancer Incidence in Saudi Arabia: 2012 Data from the Saudi Cancer Registry. *Asian Pacific journal of cancer prevention: APJCP*, *18*(9), p.2437.

Becker, S., Al Zaid, K. and Al Faris, E., 2002. Screening for somatization and depression in Saudi Arabia: a validation study of the PHQ in primary care. *The International Journal of Psychiatry in Medicine*, *32*(3), pp.271-283.

Bejjani, B.A., Lewis, R.A., Tomey, K.F., Anderson, K.L., Dueker, D.K., Jabak, M., Astle, W.F., Otterud, B., Leppert, M. and Lupski, J.R., 1998. Mutations in CYP1B1, the gene for cytochrome P4501B1, are the predominant cause of primary congenital glaucoma in Saudi Arabia. The American Journal of Human Genetics, 62(2), pp.325-333.

Bohlega, S., McLean, D., Omer, S., Al Kawi, Z., Roos, R.A., Losekoot, M. and Bakker, E., 1995. Huntington's disease in Saudi Arabia. Journal of medical genetics, 32(4), p.325.

Bohlega, S., Monies, D.M., Abulaban, A.A., Murad, H.N., Alhindi, H.N. and Meyer, B.F., 2015. Clinical and genetic features of anoctaminopathy in Saudi Arabia. *Neurosciences*, *20*(2), p.173.

Bolino, A., Muglia, M., Conforti, F.L., LeGuern, E., Salih, M.A., Georgiou, D.M., Christodoulou, K., Hausmanowa-Petrusewicz, I., Mandich, P., Schenone, A. and Gambardella, A., 2000. Charcot-Marie-Tooth type 4B is caused by mutations in the gene encoding myotubularin-related protein-2. *Nature genetics*, *25*(1), p.17.

Crankson, S.J. and Abdullah, A., 2001. Extrahepatic biliary atresia in Saudi Arabia: the importance of early diagnosis and referral. *Tropical gastroenterology: official journal of the Digestive Diseases Foundation*, *22*(1), pp.20-22.

Darraj, A., Barakat, W., Kenani, M., Shajry, R., Khawaji, A., Bakri, S., Makin, A., Mohanna, A. and Yassin, A.O., 2016. Common Eye Diseases in Children in Saudi Arabia (Jazan). *Ophthalmology and eye diseases*, *8*, pp.OED-S39055.

El Hazmi, M.A.F., Al Swailem, A.A., Al Mosa, N.A. and Al Jarallah, A.A., 2003. Prevalence of mental retardation among children in Saudi Arabia.

El Mouzan, M.I., Al Herbish, A.S., Al Salloum, A.A., Al Omer, A.A. and Qurachi, M.M., 2012. Regional prevalence of short stature in Saudi school-age children and adolescents. *The Scientific World Journal*, *2012*.

El-Awad, M.H. and Sivasankaran, S., 1992. Neural tube defects in Southwestern region of Saudi Arabia. Annals of Saudi medicine, 12(5), pp.449-452.

El-Hazmi, M.A.F. and Warsy, A.S., 1988. Glucose-6-Phosphate Dehydrogenase Deficiency in Saudi Arabia. Human heredity, 38(5), pp.317-322.

El-Sissy, A.H., El-Mashari, M.A., Bassuni, W.Y. and El-Swaayed, A.F., 2006. Aberrant lymphoid antigen expression in acute myeloid leukemia in Saudi Arabia. J Egypt Natl Canc Inst, 18(3), pp.244-9.

Ezzat, A.A., Ibrahim, E.M., Raja, M.A., Al-Sobhi, S., Rostom, A. and Stuart, R.K., 1999. Locally advanced breast cancer in Saudi Arabia: high frequency of stage III in a young population. Medical oncology, 16(2), pp.95-103.

Faheem, M., Naseer, M. I., Rasool, M., Chaudhary, A. G., Kumosani, T. A., Ilyas, A. M., ... & Jamal, H. S. (2015). Molecular genetics of human primary microcephaly: an overview. *BMC medical genomics*, *8*(1), S4.

Faiyaz-Ul-Haque, M., Zaidi, S.H., Al-Sanna, N., Alswaid, A., Momenah, T., Kaya, N., Al-Dayel, F., Bouhoaigah, I., Saliem, M., Tsui, L.C. and Teebi, A.S., 2009. A novel missense and a recurrent mutation in SLC2A10 gene of patients affected with arterial tortuosity syndrome. Atherosclerosis, 203(2), pp.466-471.

Harfi, H.A., Brismar, J., Hainau, B. and Sabbah, R., 1992, November. Partial Albinism, Immunodeficiency, and Progressive White Matter Disease: A New Primary Immunodefiency. In *Allergy and Asthma Proceedings* (Vol. 13, No. 6, pp. 321-328). OceanSide Publications, Inc.

Hassan, G.A., Taha, G.R., Mahmoud, A. and Azzam, H., 2013. Selective mutism and social anxiety disorders: are they two faces of the same coin?. *Middle East Current Psychiatry*, *20*(3), pp.156-163.

Hussein, M.M., Mooij, J.M.V., Roujouleh, H. and El-Sayed, H., 1994. Observations in a Saudi-Arabian dialysis population over a 13-year period.

Imtiaz, F., Rashed, M.S., Al-Mubarak, B., Allam, R., El-Karaksy, H., Al-Hassnan, Z., Al-Owain, M., Al-Zaidan, H., Rahbeeni, Z., Qari, A. and Meyer, B.F., 2011. Identification of mutations causing hereditary tyrosinemia type I in patients of Middle Eastern origin. Molecular genetics and metabolism, 104(4), pp.688-690.

Iqbal, M.A., Sakati, N., Nester, M. and Ozand, P., 2000. Cytogenetic diagnosis of fragile X syndrome: study of 305 suspected cases in Saudi Arabia. Annals of Saudi medicine, 20(3-4), pp.214-217.

Jastaniah, W., 2011. Epidemiology of sickle cell disease in Saudi Arabia. *Annals of Saudi medicine*, *31*(3), p.289.

Jawadi, A.H., 2010. Clubfoot management by the Ponseti technique in Saudi patients. Saudi medical journal, 31(1), pp.49-52.

Kari, J.A., 2006. Chronic renal failure in children in the western area of Saudi Arabia. Saudi Journal of Kidney Diseases and Transplantation, 17(1), p.19.

Khan, N.A., Azhar, E.I., El-Fiky, S., Madani, H.H., Abuljadial, M.A., Ashshi, A.M., Turkistani, A.M. and Hamouh, E.A., 2008. Clinical profile and outcome of hospitalized patients during first outbreak of dengue in Makkah, Saudi Arabia. *Acta tropica*, *105*(1), pp.39-44.

Khan, S. A. (2015). Epilepsy awareness in Saudi Arabia. *Neurosciences*, *20*(3), 205.

Mahmoud, A.A., Yousef, G.M., Al-Hifzi, I. and Diamandis, E.P., 2002. Cockayne syndrome in three sisters with varying clinical presentation. *American Journal of Medical Genetics Part A*, *111*(1), pp.81-85.

Malibary, H., Bucklain, I., Ayoub, O., Digani, W. and Biary, N., 2004. Dystonia and movement disorders. *Neurosciences*, *9*(1), p.S30.

Mansoor, I., Zahrani, I.H. and Aziz, S.A., 2002. Colorectal cancers in Saudi Arabia. Saudi medical journal, 23(3), pp.322-327.

Meng, L.J., Griffiths, W.J., Nazer, H., Yang, Y. and Sjövall, J., 1997. High levels of (24S)-24-hydroxycholesterol 3-sulfate, 24-glucuronide in the serum and urine of children with severe cholestatic liver disease. Journal of lipid research, 38(5), pp.926-934.

Moammar, H., Cheriyan, G., Mathew, R. and Al-Sannaa, N., 2010. Incidence and patterns of inborn errors of metabolism in the Eastern Province of Saudi Arabia, 1983-2008. *Annals of Saudi medicine*, *30*(4), p.271.

Moammar, H., Ratard, R., Cheriyan, G. and Mathew, P., 1996. Incidence and features of galactosaemia in Saudi Arabs. *Journal of inherited metabolic disease*, *19*(3), pp.331-334.

Mohamed, J.Y., Faqeih, E., Alsiddiky, A., Alshammari, M.J., Ibrahim, N.A. and Alkuraya, F.S., 2013. Mutations in MEOX1, encoding mesenchyme homeobox 1, cause Klippel-Feil anomaly. *The American Journal of Human Genetics*, *92*(1), pp.157-161.

Murshid, W. R., Jarallah, J. S., & Dad, M. I. (2000). Epidemiology of infantile hydrocephalus in Saudi Arabia: birth prevalence and associated factors. *Pediatric neurosurgery*, *32*(3), 119-123.

Murshid, W.R., 2000. Spina bifida in Saudi Arabia: is consanguinity among the parents a risk factor?. Pediatric neurosurgery, 32(1), pp.10-12.

Naeem, Z., 2015. Burden of diabetes mellitus in Saudi Arabia. *International journal of health sciences*, *9*(3), p.V.

Niazi, M.A., Al-Mazyad, A.S., Al-Husain, M.A., Al-Mofada, S.M., Al-Zamil, F.A., Khashoggi, T.Y. and Al-Eissa, Y.A., 1995. Down’s syndrome in Saudi Arabia: incidence and cytogenetics. Human heredity, 45(2), pp.65-69.

Parvari, R., Hershkovitz, E., Grossman, N., Gorodischer, R., Loeys, B., Zecic, A., Mortier, G., Gregory, S., Sharony, R., Kambouris, M. and Sakati, N., 2002. Mutation of TBCE causes hypoparathyroidism-retardation-dysmorphism and autosomal recessive Kenny-Caffey syndrome. *Nature genetics*, *32*(3), pp.448-453.

Rajapakse, C.N.A., 1987. The spectrum of rheumatic diseases in Saudi Arabia. Rheumatology, 26(1), pp.22-23.

Ramello, A., Vitale, C. and Marangella, M., 2001. Epidemiology of nephrolithiasis. Journal of nephrology, 13, pp.S45-S50.

Saggu, S., Rehman, H., Abbas, Z.K. and Ansari, A.A., 2015. Recent incidence and descriptive epidemiological survey of breast cancer in Saudi Arabia. *Saudi medical journal*, *36*(10), p.1176.

Salhia, H.O., Al-Nasser, L.A., Taher, L.S., Al-Khathaami, A.M. and El-Metwally, A.A., 2014. Systemic review of the epidemiology of autism in Arab Gulf countries. *Neurosciences*, *19*(4), p.291.

Scrimgeour, E. M. (2009). Huntington disease (chorea) in the Middle East. *Sultan Qaboos University medical journal*, *9*(1), 16.

Seyam, R.M., Bissada, N.K., Kattan, S.A., Mokhtar, A.A., Aslam, M., Fahmy, W.E., Mourad, W.A., Binmahfouz, A.A., Alzahrani, H.M. and Hanash, K.A., 2008. Changing trends in presentation, diagnosis and management of renal angiomyolipoma: comparison of sporadic and tuberous sclerosis complex-associated forms. Urology, 72(5), pp.1077-1082.

Taylor, J.W., 1963. Cancer in Saudi Arabia. *Cancer*, *16*(12), pp.1530-1536.

Twigg, S.R., Lloyd, D., Jenkins, D., Elçioglu, N.E., Cooper, C.D., Al-Sannaa, N., Annagür, A., Gillessen-Kaesbach, G., Hüning, I., Knight, S.J. and Goodship, J.A., 2012. Mutations in multidomain protein MEGF8 identify a Carpenter syndrome subtype associated with defective lateralization. *The American Journal of Human Genetics*, *91*(5), pp.897-905.

Yaqub, B.A. and Daif, A.K., 1988. Multiple sclerosis in Saudi Arabia. Neurology, 38(4), pp.621-621.

Zaidi, S.H.E., Faiyaz-Ul-Haque, M., Shuaib, T., Balobaid, A., Rahbeeni, Z., Abalkhail, H., Al-Abdullatif, A., Al-Hassnan, Z., Peltekova, I. and Al-Owain, M., 2012. Clinical and molecular findings of 13 families from Saudi Arabia and a family from Sudan with homocystinuria. *Clinical genetics*, *81*(6), pp.563-570.

Table S3. List of responses and categorization of the advantages and disadvantages of prenatal diagnosis.

Advantages Categories:

1. Early diagnosis and correction of genetic abnormalities;
2. Awareness and medical planning;
3. The choice of abortion;
4. Psychological readiness;

Disadvantages categories:

5. Psychological pressure.
6. Invasive procedure;
7. Inaccurate diagnosis;
8. Against faith;
9. Expensive;
10. The choice of abortion.

**Translation of the entries from respondence for advantages of prenatal diagnosis**	**Translation of the entries from respondence for disadvantages of prenatal diagnosis**	**Advantages categories**	**Disadvantages categories**
To know the physical health and that it is disease free. And to take precautions and perform early treatments before the disease develop.		1	
Take precautions and be mentally prepared		4	
Find future solutions		2	
Mental preparation to accept it		4	
It helps a lot to take a proper decision whether to terminate or continue.		2,3	
Mentally prepared and a choice to abort if you are not against it		3,4	
To decrease the percentage of deformed children, abortion		1,3	

	Couldn't find an advantage. I lost a boy and a girl due to health and ministry. My other two sons got cured in Germany. I have hope so please change the current status.		6
To handle and address the disease early		1	
Raise awareness		2	
Avoid problems for the child		1	
Know if the fetus is healthy or not		1	
To understand in advance any health risk of newborn child		1	
Know the type of disorder and the percentage for it to be cured		1	
Decrease diseases and pregnancies for the family and to avoid things that leads to disease.		2	
Know diagnosis in advance and plan to avoid that in future	Depends on the method used	1,2	6
Avoid problems by treating it or to leave the marriage with the person that has disease		1,2	
Chance to contemplate about the life difficulties of the child and the family and to know the degree of acceptance and ability to deal with it		2,4	
Makes the pregnant woman rest assured about the safety of the patient		1	
Mental preparedness of the family before the birth		4	
Discover the disorder before completing the pregnancy		1	
Discover the disorder		1	
May help cure the disease		1	

To protect the child and his family from the consequences of the disease and the negativity of society		1,	
Early treatment and prevent birth risks and I don't see any disadvantages		1	
To prepare the parents for whatever disability their baby has		2	
Know if there are risks and to go for abortion	Do examination for a healthy child	3	6
To handle the situation before birth and be mentally prepared to accept the child after birth		1,4	
Precaution to prevent a life that satisfies no one even the patient		1	
To diagnose earlier the genetic disorders and do interventions early		1	
To diagnose earlier the genetic disorders and do interventions early		1	
To mentally prepare the parents. And to provide needed care for the child. And decide to give birth or not and I don't see any disadvantages		3,4	
Important to prevent potential negatives. And to prevent mental shock for the parents if their baby has a disease.		2,4	
Protects people from chronic fetal diseases and saves them from hardships.		1	
The advantages include being able to diagnose serious disorders before the baby reaches a critical age, this will give parents the opportunity whether they want this kid or not.		1,3	
	Performing it half through pregnancy or at the end of it		7
Know the type of disorder the fetus has		1	
Cure the disease or undergoing abortion and mentally prepare		3,4	

the family			
Do treatment plan		2	
take precautions		2	
To mentally prepare the mother and to have the choice of abortion. And to give the mother a chance to know how to deal with the situation and her child.	mental shock for the parents and especially the mother and makes her very scared of the next step and the new situation	2,3	5
To mentally prepare the parents to welcome the child and to search for specialized health centers		2,4	
To mentally prepare the parents to welcome the child and to search for specialized health centers		2,4	
To decrease the number of children born with genetic diseases		1	
To know how to deal with patient		2	
Mentally prepare parents before giving birth		4	
	I don't trust it very much		7
To know the medical condition and treat it		1	
To decrease the number of children born with genetic or chronic diseases		3	
Precaution from diseases or deformities		2	
To abort the fetus		3	
early diagnosis is important		1	
	It causes abortion most of the time		6
Mental preparedness		4	
Early diagnosis of diseases or deformities		1	
To cure the condition or decrease its symptoms. And mentally prepare parents and educate them on how to deal with chronic		2,4	

diseases.			
Early intervention	more stress	1	5
That there is medical or surgical intervention	if there is no cure	1	5
Decrease diseases		2	
Precautions, early treatment and care		1,2	
To handle the situation early		1	
Mentally prepare parents		4	
To consider abortion		3	
Decrease the mental shock and hardships for the parents and child, especially children with mental diseases		4	
Know the diagnosis before birth and try to mitigate the disease as much as possible		1	
Checking up on the mother's and baby's health		1	
Treatment before birth if it exists	It affects the mother's mental health	1	5
good for diagnosis		1	
Decision regarding continuation or not. And delivery		3	
Prepare parents for the situation and to treat the condition		2,4	
Medical abortion option		3	
To prepared for a sick child and to find treatment methods. And to adapt to the situation		2,4	
Gives the family a chance to take proper decisions regarding the child	psychological stress for the mother and family	2,4	
Early diagnosis		1	
Know the problem and possible solutions		1	
It helps to avoid complications		1	
Psychological			5
Mental preparedness		4	

Decrease deformities		1	
To mentally prepare the mother and to know if she's capable of taking care of the child or not. And to know how to take care of the child.		2,4	
		1	
Possibility of abortion		3	
To treat the condition		1,2	
To abort the fetus		3	
To treat the child and modify its genes and having the chance of abortion		1,3	
To mentally prepare parents to know the causes of the disease		1,4	
Early diagnosis		1	
Early diagnosis		1	
Early diagnosis		1	
Identify diseases		1	
The decision to abort the child or not		2,3	
Early treatment before birth		1	
The decision to abort the child or not		3	
To mentally prepare the mother		4	
to know the child is healthy or not and decide to continue the pregnancy or not		1,3	
to mentally and physically prepare the parents so they provide the appropriate environment for the child		2,4	
Knowing if the fetus is affected before delivery and mentally prepare for it. -: risk of abortion with the amniocentesis, even if the fetus is affected will not do abortion(forbidden religiously)		1,4	6
Decreased suffering for the mother and early diagnosis which decreases the stress and hardships for the mother		1,4	

To mentally prepare parents to welcome a disabled child		4	
To be prepared and gain knowledge and learn more about the disease	frustration for the family and anxiety	2	5
To know the type and severity of the disease and treat it if needed		1	
To know the disease and be prepared	the desire of parents to abort the child	1,2	10
Before the 120 days, it would give the parents the freedom to decide whether to keep the baby or not, and prepare the parents		2,3	
Preventing your child from living with comorbidities		3	
To treat what should be treated		1	
Mental preparation		4	
Chance to abort the sick fetus	Psychological pressure and abortion risk	3	5,6
	against religion and inaccurate diagnosis		7,8
Awareness and Preparation for treatment		2	
I don't want sick kids		3	
Prepare the parent psychologically prior to delivery, giving the chance for the medical termination of pregnancy, increase the awareness of pre-marriage genetic screening e		3,4	
Preparation for the newborn needs	Psychological pressure and against religion	2,	5,8
To be ready		2	
Help to create a healthy new generation free of genetic diseases		1	
To reduce the birth of desisted infants		1	

Avoid having a child with a disability		3	
	Negative impact on the mother		5
Avoid some diseases		1	
treating it early before birth		1	
Discovering the disease and trying to treat it		1	
Being aware about the health situation of the baby	overthinking of the baby’s health until delivery	1	5
knowing if the fetus has a disease		1	
early treatment		1	
parents preparation		4	
mentally preparation and early treatment		1,4	
early treatment		1	
protect the child and the mother		1	
mentally preparation of the mother		4	
Parents have the option of abortions		3	
mentally predation of parents, abortion if serious disease		3,4	
It’s an excellent tools, provide specific diagnosis and can offer termination if affected non compatible with life		1,3	
preparation for receiving an affected child and how to deal with him		2	
	The principle of Sharia is not abortion		8

preparing for the condition	increase the probability of abortion	2	6
abortion		3	
Determine the status of the baby and what he needs according to his health status		2	
abortion		3	
Reduce the chances of getting a genetic disease or passing it on to children		1	
Reduce the transmission of genetic diseases to children		1	
Provide the necessary health care		2	
early diagnosis		1	
early diagnosis	stress and anxiety after the diagnosis	1	5
decrease the complications		1	
early treatment	The diagnosis might be inaccurate	1	7
Prevention		1	
to diagnose the fetus		1	
diagnosis and to know how to treat the fetus		1	
preparing the mother for her affected child		2,4	
Check on the health status of the fetus		1	
abortion	negative impact on the family	3	5
Being aware about the health situation of the baby. Early diagnosis and treatment	Psychological pressure	1	5

mentally preparation		4	
early treatment		1	6
To be prepared for any complications during deliver , Help the parents to cope with the disease and have an idea about it before the baby is born		2	
	expensive, stressful, might lead to miscarriage		6,9
early diagnosis for sever diseases and having the choice of abortion		1,3	
	complications for the mother and the child		6
disease treatment		1	
early treatment , medical planning and mentally preparation of the parents		1, 4	
decreasing the birth of children with disabilities / the possibility of early treatment		1	
mentally preparation or abortion		3,4	
to make the decision to keep the pregnancy or not		3	
abortion	the difficulty of taking the abortion decision, against faith	3	5,8
	anxiety during the pregnancy		5
Reduce the suffering of the patient and his family.		3	
There might be some medical measures that can be done before birth or immediately after birth	anxiety during the pregnancy	1	5
to avoid the disease and its consequences		1,2	

Treatment can be done during pregnancy, and abortion if the deformation is severe		1,3	
for precautions		2	
		1	
to avoid having an affected child		3	
early treatment		1	
to decrease the abnormal cases		1	
if there is chance for abortion	error or and invasive	3	6,7
if there is chance for abortion	error or and invasive	3	6,7
early treatment		1	
decrease the genetic diseases		1	
to know the genetic diseases and how to treat them		2	
To know the options of the treatment and the prognosis of the disease		2	
Avoid negative consequence and mitigate risk of quality of life		1	
Avoid the birth of an affected child		3	
To prevent recurrence		1	
Prevention of disease, choosing healthy child with HLA matched donor for affected child who needs SCT.		1	
abortion and mentally preparation		3,4	

mental preparation of the family		4	
	Infection, abortion		6
	inaccurate		7
prevent the genetic diseases		1	
to know the health status of the fetus		1	
Early diagnosis and treatment. Mentally preparation		1,4	
Identifying the genetic defects and the treatment	Abortion and it can be harmful to the mother	1	6
To avoid consequences		1	
mentally preparation for the disease and its needs		2,4	
early treatment	negative impact on the mother	1	5
Early diagnosis	causing stress to the family	1	5
preparation of the family		4	
mentally preparation		4	
parents preparation		2,4	
to find a treatment or abortion		1,3	
to know the symptoms of the disease before it occurs		1	
Protect the child from living a life full of suffering and pain		1,3	
prevent spread of the disease		1	
to know the disease and how to avoid it	mental stress for the parents	1	5

for fetus safety		1	
prenatal diagnosis will help to solve some of the problems of the disease		1	
preparation for receiving the newborn or discussing abortion		2,3	
Choice of abortion if possible. If not, Psychological readiness	Sadness, anxiety	3,4	5
Prepare the parents with a way to deal with the child's abnormality.	Parents may become depressed	2	5
parents preparation for the affected child		4	
early detection of the genetic disease	it might be harmful to the fetus	1	6
PGD lets know the genetic disease before fetal development & provide the ability to terminate pregnancy earlier, & to choose the healthy zygote.	it can cause trauma if all fetuses affected	1,3	5
reassurance, might consider abortion if fetus is affected		1,3	
To do abortion in early pregnancy		3	
important to know the disease and the treatment or abortion if needed		1,3	
decrease the consequences		1	
When advised that my baby is an SMA patient I may request abortion if age is less than 120 days.		3	
To know in advance and maybe have an abortion		1,3	
to know the disease before birth and to prepare for it		1,2	
to know the diseases		1	
Treatment of the diseases is or minimize it /child's abortion		1,3	
Prevention and early intervention		1	

preparation and taking the final decision on having child or not		3,4	
mental preparation of the parents	the mother will be in pain during analysis	4	6
Diagnosis and treatment of certain diseases as well as abortion of the fetus if it has a sever diseases		1,3	
	Inaccuracy in results		7
Avoidance of disability		1	
abortion for the affected fetus		3	
Parents have the option to complete the pregnancy or not , and psychological and physical preparation		3,4	
to know the disease before birth	The pregnant mother will remain in stress condition during pregnancy	1	5
Take the necessary precautions and make a decision to keep the pregnancy or not		2,3	
Complete pregnancy in the case of healthy fetus / get rid of the pregnancy if the fetus is affected		3	
to prepare the parents		2	
mental and medical preparations to receive the affected child and immediate care after birth	Suggestion of abortion is absolutely unacceptable	2,4	8
The parents can decide about their child who will be born disabled, and have a decision for abortion		3	
to Know and eliminate any disease that the fetus has		1	
Early detection and trying to find a treatment if possible and early preparation.		1,2	
The chance to choose between keeping the baby or losing it as well as considering an early intervention approach		1,3	

to know the genetic diseases		1	
to know the genetic diseases and their prevention		1,2	
Early detection which helps to take appropriate medical intervention	may adversely affect the mental status of pregnant women, or the relationship of the family	1	5
Prevent birth of genetically affected generation on future		3	
Be prepared and find proper treatment		2,4	
mental preparation at least		4	
Make a decision to continue or terminate the pregnancy		3	
It is very important to know the health of the fetus		1,2	
Avoid having a child with a disability		3	
Avoid diseases		2	
preparation of the parents and trying to understand the disease	the mental state of the pregnant mother	2	5
The mental preparation to accept the disease... abortion in accordance with Sharia	Psychological pressure, Some of the diagnoses are expensive	3,4	5,9
Avoiding problems in advance and preparing parents for future responsibilities and better treatment opportunities		1,2	
mentally preparation of the child	psychological trauma to parents	4	5
The mother is mentally prepared	unexpected reaction	4	5
abortion of the fetus, Childbirth in an advanced hospital		,3	

Avoiding the birth of an affected child.	the psychological crisis and confusion in which the parents are determined to determine the fate of the fetus	3	5
treatment		1	
Avoiding having of children with disabilities	abortion which is against god willingness	1	8
to know the condition of the fetus and make a decision to complete the pregnancy or not		1,3	
to know the condition of the fetus and try to treat what can be treated before birth		1	
abortion of the fetus at an early stage	anxiety	3	5
To reduce the prevalence of handicapped children		1	
Minimize births with genetic diseases		1	
mother mentally preparation and to choose whether to keep the child or to abort it		3,4	
Early dx and action		1	
The possibility of avoiding the consequences	stress and anxiety of the pregnant woman during the pregnancy period, the possibility of an error in the diagnosis	1	5,7
to know the disease and what percentage of having an affected children		1,2	
	The diagnosis might causes abortion		6

Identifying the disease, ending the pregnancy and ending the suffering of the parents		1,3	
To reduce the symptoms of the disease or treat it if possible		1	
To know and avoid any diseases		1	
Good timing for termination		3	
decrease some diseases in the family by preventing the birth of the affected children		1,3	
To prepare the parents		2	
To prevent or decrease the effect of the disease as much as possible		1	
early diagnosis and rapid intervention		1	
to prepare and accept the child		2,4	
Allows the mother and father to be prepared and to gain information about the disease		2,4	
to be prepared for the problems that occur after birth		2	
Avoid the genetic diseases in children to live a normal life		1	
mentally preparation for parents and to increase the possibility of treating the child or creating a suitable environment for him		2,4	
In order to be aware of the state of health of the child and be prepared psychologically to accept the situation and to treat the child immediately		1,4	
	Mother will continue the Pregnancy with much stress		5
to avoid the complications		1	
To know the condition of the fetus and its safety from any		1	

disease			
to know the diseases		1	
Protects against the spread of the disease		1	
Prevention of the disease	increase in accidental abortion in negative cases	1	7
		1	
to prepare for receiving the affected child and create a good atmosphere for him after birth		2,4	
to know the diseases and avoid them early		1	
Treat the fetus as much as possible before birth		1	
treat any disease before birth		1	
Avoiding diseases		1	
it avoids the parents from having an affected child		3	
Sometimes consequences can be prevented		1	
mentally preparation before birth and more education about the disease, abortion	psychological pain and depression	3,4	5
Pregnant woman will not be surprised by her child's abnormality and she will be prepared for it	mother mentally disturbed and she will need time to overcome this situation and affect the fetus	4	5
Knowing if the a fetus has a disease or a defect and early detection	if it is not a cure treatment would be a bad mental state of mind	1	5
For prevent any psychological shock to the family for the next deliveries and to know how to deal with that and to prepare		2,4	

for the mother safety and parent's awareness	the presence of a affected child	2	5
Early diagnosis to have a healthy children		1	
The family can prepares for the child and provide the therapeutic requirements before birth.	the trauma that mother can have during the pregnancy	2	5
socially to decrease the spread of genetic diseases		1	9
to complete the pregnancy or abortion		3	
delivery plan in a good medical center,		2	
early diagnosis		1	
mentally preparation		1,4	
to avoid the disease and control it from the beginning		1,2	
possible to find a solution or treatment for the fetus		1	
Preparation for delivery as indicated + contraception or ivf		2	
Avoiding the diseases for both fetus and the pregnant women		1	
Reduce the incidence of genetic diseases in newborns		1	
To treat the disease before it becomes more severe. Second: Preparing the parents and the family and preparing the environment to receive the child with any genetic or hereditary disease		1,2	
Avoid the disease at birth		1	
To avoid the problems in children after birth		1	

early diagnosis		1	
knowing of the diseases and the possibility of treatment before birth		1	
The family preparation for their son's condition, helping for the treatment early		2,4	
The possibility of abortion in early diagnosis, awareness and follow-up		2,3	
to Know the disease and discuss the necessary measures		1,2	
the right action before the birth		1	
mentally preparation to accept the condition of the fetus in case of the disease		4	
There may be something that can be corrected before birth		1	
To identify the disease and identify the appropriate diagnosis and treatment or to take the right decision		1,3	
In order to prepare for the child according to his / her condition		2	
		1	
Avoid the diseases before it happen	mentally stress of the parents	1	5
Early management, search for high center to manage baby ASAP		1,2	
Earlier diagnosis & management is the main advantage.	Disadvantage higher abortion rate	1	8
mentally preparation and prepare the appropriate environment for the child after birth		2,4	
Early diagnosis gives the parents an option to keep the fetus or not		1,3	

Termination or treatment		1,3	
Important to reduce risks		1,2	
Discover the disease and try to treat it		1	
Avoid possible complications		1	
Reduces the proportion of affected child		1	
Knowing the safety of the fetus and the genetic diseases		1	
It is considered as preventive measures.		2	
Parents preparation and to know how to take care of the disease		2,4	
helpful In some cases		1	
Avoid most of the disease during the Pregnancy , knowing the diseases and early diagnosis		1	
Parents must know about the disease and prevent the shock before the birth of the child and be mentally preparation		1,4	
therapeutic intervention depends on the type of genetic disease		1	
The birth of healthy children, the prediction of disease and the possibility of treatment	the vulnerability to the mother, the death of the fetus	1	6
mental preparation for the birth of a affected child	abortion which against god willingness and constant anxiety	4	5,8
reduce disability and seek early treatment, or decide on abortion		1,3	
Control the disease and possible treatment		1	
disease can be treated better		1	

to find a proper way to treat fetus		1	
Take proper ways to treat the disease		1	
To avoid diseases and disabilities		1	
to check or identify the type of the disease tend to provide the treatment early		1,2	
to be aware and avoid any harm before birth		1	
to know the condition of the fetus and to treat the disease		1,2	
Treatment if it is possible		1	
Helps to treat early		1	
prepare the appropriate environment for the disease,	the negative concerns parents	2	5
Is very necessary to avoid future disabilities		1	
better to know before the child birth		1	
Knowing the disease early		1	
Protection from pregnancy with an affected child		2	
	mother might think to do for abortion		8
Avoiding genetic diseases		1	
To ensure the health state of the fetus and the mother		1	
Some diseases can be treated in the case of the fetus and may extend to prevent a disability		1	
Preparing for any disability or chronic illness of the fetus		2,4	
early treatment for both the mother and the fetus		1	

The discovery of the disease of the fetus before birth and the choice of parents either by abortion or continuation of pregnancy		1,3	
Knowing the disease	the mother will lives in a bad mental state	1	5
for prenatal treatment		1	
For the mentally preparation of the parents to accept child		4	
Understanding of fetal status and parents preparation and planning for the next procedure	the diagnosis may not be accurate	2,4	7
Save the fetal life and avoid genetic diseases		1	
Abortion. Avoid pregnancy with another infected child		2,3	
good for parents prefer abortion		3	8
to decrease the incidence of affected children in the society		1	
To reduce its risk and find a treatment		1	
to treat the disease early		1	
To identify and treat the disease and avoid its risk		1	
Giving the parents the option to keep the fetus or abort it, the mental preparation of the parents		3,4	
abortion the affected child		3	
Very useful to avoid the risks of disabilities and genetic diseases		1	
Early detection of some diseases which can be alleviated by early intervention		1	
Avoiding genetic diseases		1	
Reduces the incidence		1	

Preparing the family to receive the child	child might not be accepted by the parents	2,4	5
Healthy offspring		1	
to avoid any disease after child birth	The fetus may be injured during the diagnosis	1	6
prepare the mother and the father psychologically and scientifically to deal with the child		2,4	
Prenatal diagnosis makes it easier to deal with the condition		1	
Avoid pregnancy completion or treatment before birth if possible		1,3	
Find solutions to the problems		1	
Diagnosis protects against disease		1	
The mother can get rid of her fetus		3	
Preparing the parents mentally to receive the affected child		4	
Some diseases can be prevented before delivery		1	
Awareness the parents about the disease		2	
The possibility of early intervention and treatment	mental state of the mother	1	5
Useful to avoid deterioration of the situation		1	
Knowing how to handle the affected child and be more careful if the mother will be pregnant again		2	
Take action during pregnancy, at birth and after birth. Psychological and physical readiness and the creation of a suitable environment for the child and the mother. Parents accept their child before birth to avoid trauma		2,4	

The mother can abort the fetus		3	
The parents have the knowledge from the beginning of the pregnancy whether the child is healthy or affected and thus easy to respond to the situation		1,2	
To find out if there is a hereditary disease and avoid them		1	
early detection of the genetic disease	Some errors that may kill a mother or fetus	1	6
education and preparing the environment and searching for service centers		2	
Psychological, environmental and educational preparedness and to find ways to reduce the complications		2,4	
early treatment	the mental state of the mother	1	5
To know about the fetus health		1	
	I do not find justification for the diagnosis		8
therapeutic intervention depends on the type of genetic disease		1	
So that we have the choice between aborting the fetus or not		3	
Identification of deformity and genetic diseases		1	
To know about the fetus's disease		1	
Parents' mentally preparation for what they will face and teach them how to deal with children with genetic diseases.		2,4	
saving the mother and fetus as possible		1	
to avoid repeating children with hereditary diseases		1	

to prevent and avoid the genetic diseases		1	
to be assured of child health		1	
treatment	the mother will be worried	1	5
preparation		2,4	
mentally preparation for the arrival of the affected child	direction of abortion	4	10
Helps to treat the disease early before the complications		1	
Treatment of genetic diseases in the early stage		1	
It is beneficial to those who wish to abort the fetus because of his disability;	but it is prohibited by sharee'ah	3	8
Avoid disabilities, malformations and diseases		1	
Avoid genetic diseases as much as possible or detect them early		1	
Helps the mother to know what the child has and the best for his condition		1,2	
It is very important to help the mother & her fetus if possible.		1	
Early reassurance on child health.	Length of diagnosis and examination	1	5
treatment of the fetus and saving the mother	mother may know about the disease of her fetus and affect her mental state	1	5
	Continuing anxiety		5
Avoiding genetic diseases		1	
To know the disease and treat it		1	
better to avoid too many risks and consequences		1	

Avoiding genetic diseases	Inability to prevent diseases if there are prenatal problems	1	8
to know the problem and help as much as possible in reducing the impact on the life of the baby		1	
Predicting and treating diseases in advance		1	
It is best to diagnose if there is a treatment		1	
Educate both mother and family about dealing with such diseases		2	
Early treatment	mental state of the mother	1	5
Treatment of some diseases is possible	stress	1	5
	even if the diagnosis is performed before the birth, it is not permissible to abort the fetus		8
Know the type of disease and treatment or psychological preparation to receive that child	negative impact on the mental health of the pregnant woman	1,4	5
treat the fetus that it is possible or make it less harmful to it		1	
Early detection of diseases and malformations		1	
Save the mother from raising a disabled child		1	
take the action before its happens		1	
Reduce the birth of disabled children		1	
Knowing the child's safety, the type of disease, the symptoms, and the most important is the abortion of the child if it is necessary		1,3	

To know the fetus's safety		1	
option for the parents to abort or not		3	
to reduce the transmission of the genetic diseases		1	
mentally preparation		4	
knowing the type of disease that children will have and how to avoid these diseases		1	
To avoid the genetic diseases and pregnancy problems		1	
Terminate per. If possible		3	
Precaution or possible treatment plan		2	
early diagnosis and intervention		1	
decrease the genetic diseases	The treatment methods	1	
mentally preparation of the mother and treatment before birth		1,4	
early diagnosis and abortion		1,3	
Reducing the genetic diseases		1	
mentally preparing of the parents to reduce the impact of a disease and find out the possibility of the treatment		2,4	
mentally preparation of the parents		4	
ensure the safety of the fetus at the beginning of pregnancy and maintain it		1	
avoid having affected children		1	
Abortion can done if the fetus is very ill	Psychological pressure on the parents during the pregnancy and after delivering	3	5

Preparing the parents to receive an abnormal child / abortion, which will reduce the cost on both government and the parents	may cause tension and anxiety	3,4	5,7
the parents will be aware about the fetus so they can decide abortion in case of abnormality		3	
to know about the health status of the fetus		1	
	it hurts the parents and they can do nothing to the child		5
diagnosis of the disease		1	
Early treatment, abortion		1,3	
Possibility to treat certain diseases early		1	
to know what is the abnormalities of the fetus		1	
helps the parents to develop an idea about the situation		2	
to know what disease is affected the child and its causes		1	
preventing from having an abnormal child		1	
to increase parents awareness		2	
Avoid having diseased children with no treatment available		1	
for preparation		2,4	
Avoiding to have an affected child		1	
Avoid most of the diseases		1	
very good to avoid genetic diseases		1	

Diagnosis of the fetus and preventing the recurrence of genetic diseases in the family	Abortion, inaccuracies, lack of advanced laboratories for genetic analysis, issuing the result of sample	1	10,7
mentally preparation for the affected child	Self-frustration	4	5
no doubt, it is excellent for treating the abnormalities		1	
Good for mentally preparation and to learn how to deal with the affected child		2,4	
Early diagnosis so it can be treated		1	
diagnosis the disease early		1	
Mentally preparation for the next stage.	Living with anxiety and conflict	4	5
The possibilities of early treatment	Needs skilled medical team	1	6
	It might be harmful to the mother		6
Detection of the disease and how to treat it	Tithe mother will be stressed during her pregnancy	1	5
Mentally preparation for future conditions	Causes anxiety and thinking of abortion	4	5
Intervention before the birth of a disabled child		1	
Mentally preparation of the parents		4	
To know the fetal status and the possible of treatment before delivery		1	

Decrease the incidence of an infected child and also abortion will reduce the prevalence of the disease in the society		1,3	
To check health status of the fetus		1	
Correcting the problem, treatment		1	
The mother can know about her fetus in advance before birth	Psychological pressure	1	5
Faster treatment of the disease		1	
Early treatment		1	
Saving the fetus's life or treating it		1	
To know the disease	abortion due to the method of examination	1	6
To Know the disease and find solutions for it		1	
Learning how to live with a child who have abnormality and prepare for it		2,4	
Knowing the genetic diseases that affect the child and preparing them before birth and also termination of pregnancy is if the diseases is very severe		2,3	
Protecting the mother and fetus from anything that may affect them by correcting it before birth.	side effects of the procedure on the mother and fetus	1	6
Mentally preparation for the condition	stressed mother during the pregnancy	4	5
Helps the mother and father to know what they expect	The sadness when the child is affected	2	5
The possibility of treatment.to avoid the consequences of the disease		1	

Early detection of diseases and how to deal with it		1,4	
treatment before birth		1	
Early prevention	Stressed parents	1	5
The discovery of diseases early, which gives the opportunity for early treatment	the negative is the presence of errors in the diagnosis and inaccuracy	1	7
Planning to continue the journey of pregnancy		2	
Helps us to prevent this disseizes		1	
Treat some of the diseases before the child birth		1	
Early intervention before it's too late		1	
Parental awareness of the situation and psychological preparation to accept the disease		2,4	
There might be a treatment or intervention in the right time		1	
Treatment during pregnancy		1	
It helps the mother from having affected child		1	
Treatment if possible and mentally preparation for the child		1,4	
Treat the fetus if it’s possible or terminate the pregnancy		1,3	
Treatment		1	
Having a healthy child free from diseases		1	
We can help the health of the mother and baby by getting the diagnosis.		1	

Detection of the diseases	inaccuracy in diagnosis	1	
Avoiding risks for the mother and fetus		1	
Counselling for parents, chances for therapeutic abortion, any way for managements either early or late during pregnancy, or even during labor.		2,3	
	mother feelings		5
Mentally prepare patients		4	
To know the problem before it occurs and to do abortion		1,3	
If there is chance for intervention and treatment before or immediately after birth	Parents will get stressed	1	5
Mental and medical preparation		2,4	
Avoid genetic diseases in all their forms		1	
Find out the condition of the fetus early		1	
Take caution of genetic and hereditary diseases		2	
Avoid birth defects and cure diseases		1	
Avoiding child suffering and treatment if possible		1	
Prenatal diagnosis makes it easier to deal with the condition		1,2	
Help to avoid the diseases		1	
It can help the treatment of the fetus		1	
To know if there is any health problem in the fetus and start to treat it before it is too late		1	
Early reassurance of child health.	Length of diagnosis and examination	1	5
Detection and treatment of genetic diseases		1	
To know the health condition of the fetus and if it has deformities or health problems		1	

Helps solve many problems after childbirth		1	
The preparation of parents and especially the mother to exceed the severe grief during the treatment of the child after childbirth		4	
The mother can takes the necessary measures and treatment during pregnancy and not shocked at birth		2,4	
	Mother will have anxiety which affects the health of her and the child and our religion prohibits the abortion of the fetus no matter what degree of deformity		5,8
To reduce the diseases and disabilities in the community		1	
Protect the child and family from illness		1	
To know the possibility of the treatment		1	
Reduce the chance of the disease		1	
Treatment of the disease if possible, and prepare the parents		1,2	
Early intervention in treatment or abortion		1,3	
Not having a child with a disability		1	
Discover the problem from the beginning and solve it if possible		1	
Treatment and avoidance the diseases	diagnosis error	1	7
To reduce the birth of people with incurable diseases and avoid the mentally pain is the most important		1,4	
to avoid complication before birth		1	

Knowing of diseases that can be avoided		1	
The parents preparation to their child		2	
Parents are mentally prepared, and if there is any treatment or need for a special help	the anxiety and sadness that afflicts the parents	1,4	5
Helps to avoid or treat any abnormalities early		1	
For prevention and awareness		2	
Preventing disease and helping to understand the disease for the parents		2	
Mentally prepared	Mother can decide to go for abortion under the stress	4	5
	very expensive		9
To know how to deal with this disease		2	
Abortion in the event of a disability		3	
Avoid the diseases		1	
Knowing about the child safety		1	
I think that it is necessary for parents to know the state of the fetus health early so that they can make decisions or prepare for it		2,3	
Diagnosis of the fetus before birth in order to avoid abnormality		1	
Preparing and finding ways to deal with the child	Stress	2	5
To know what you might face and to reading about the disease and find solutions if any		2	
To ensure the safety of the fetus or if there are any diseases, the parents are ready for full care and satisfaction with fate and destiny		1,2	
Be more careful, take care of my nutrition		2	

To better take decision and to better prepare yourself for what's coming.	Although knowing the truth might be hard	2	5
Helping parents and reducing the risks		1,2	
Avoiding the disease		1	
The ability to get rid of the fetus if it is diagnosed with any disease		3	
Psychologically preparation		4	
early treatment		1	
It is very important to diagnose to know the infected cases and get rid of the fetus early and to purify a person from life by torment and to prevent parents from the difficulties of caring for disabled children		3	
Preparing and Accepting of the Child, Early Treatment	Depression	1,4	5
To know the problem and solve it		1	
More options for mother and father prepare for the problems ,abortion and intrauterine treatment		2,3	
Abortion		3	
	The stress state of the parents		5
	The negative impact on the mother 's child		5
Limits the spread of genetic diseases		1	

If there is treatment of the child during pregnancy it is wonderful to diagnose	makes the mother in a state of stress during the months of pregnancy and it may be wrong diagnosis after all this effort through an experience I had before	1	5,7
	Sometimes the diagnosis is not like what they think		7
Psychological preparation of parents before birth / education about the disease and how to deal with it during pregnancy and after birth		2,4	
For treatment or follow-up or abortion in case of severe deformation		1,3	
Ensure fetal safety		1	
Prevention and treatment		1	
The discovery of the disease and treatment and to avoid complications		1	
disease detection	possible harm to the fetus	1	6
the possibility of treating some defects and diseases within the uterus and also can be aborted of before the birth	of course the possibility of abortion	1,3	6
Take the right decision for mother and child		2	
To avoid the bad situation		2	
To know what is going on before birth		2	
For parents to plan about accepting the child or ending it from		2,3	

the beginning			
	Psychological deterioration		5
Learn about the possible diseases and the ability to treat them early		1	
The family accepts the disease of the child		4	
Psychological readiness		4	
If it is possible to treat the fetus		1	
Helps with early treatment or abortion if this is important for the life of the mother or fetus		1,3	
preparing before the surprise after the birth	Psychological pressure of the mother	4	5
having a healthy children		1	
Medical intervention to save the fetus from genetic diseases		1	
	They may diagnose him as sick and God willing that the child will be born healthy		7
Avoid what can be avoided before giving birth		1	
Early treatment		1	
Educating the parents		2	
Detection of some genetic diseases		1	
Avoiding new diseases and disabilities in the society which is difficult to treat		1	
The possibility of medical intervention such as the operation of the fetus when it’s still inside the uterus	It may affect the life of the fetus, success is always not guaranteed.	1	6

For the benefit of the mother and early treatment		1	
The readiness of the mother and giving the child a greater care		2,4	
Being able to deal with the problem early		1	
The disease can be prevented by early treatment or by reducing the risk of the disease		1	
Prepare for the challenges		2	
	The difficulty of treatment, needs great accuracy		7
For parents to plan either by accepting the child or ending it from the beginning		2,3	
Advance information		1	
Early diagnosis		1	
preparing the parents and to know the possibility of treatment and to reduce the impact of the disease		2,4	
To avoid any problem during pregnancy and to find a suitable solution		1	
Detection of genetic diseases		1	
Prevention and treatment from the beginning of the disease		1	
Reduce the incidence of genetic diseases or even the birth of abnormal children no matter how painful the results		1	
Ensure the safety of the fetus		1	
Identification of genetic diseases and disabilities		1	
Helps to know the child's diseases and how to treat them	the prenatal diagnosis is not safe	1	6

To know the diseases and treat them early and to know the sex of the child		1	
Identify the diseases before birth and try to treat it		1	
avoiding genetic diseases so the children can live normally		1	
The advantages of prenatal diagnosis are many, including the discovery and prevention of diseases and problems related to the child early		1	
To know the medical problem and solve it		1	
	after diagnosis ,the mother might go for abortion when she discover the disease because she don't want affected child		10
To do what is possible early		2	
avoiding genetic diseases		1	
avoiding the genetic diseases and avoiding health problems during pregnancy		1	
Avoiding problems in the future		1	
Get the chance to terminate pregnancy if the fetus is abnormal	lack of Accuracy	3	7
Aborting the affected fetus in the right time		3	
Check on the status of the fetus		1	
prevention of the diseases		1	
In order increase the parents awareness about the disease	parents can decide to abandon the fetus illegally	2	8

Parents will know what difficulties they might face in caring for their child and take the precautions and procedures necessary to care for their child properly		2	
Provides you with information about the diagnosis and the treatment of the condition before birth		1	
Knowing the fetus if it has a disease and therefore the idea of abortion is on the table	psychological aspects	1,3	5
Early intervention		1	
To prepare the parents psychologically and to educate them in all psychological aspects, which they must know to grow the child in a healthy way		2,4	
Have a good knowledge about what to expect and be prepared		2	
Psychological readiness		4	
It is possible to be in an early way of treatment and must be prepared for it	stress and sadness in the period of pregnancy and which is harmful	1	5
Maybe can know how can get less disease or avoid it		1	
For the possibility of early intervention and early treatment of the condition or to terminate the pregnancy if it is serious		1,3	
A decision might be taken at the proper time		1	
	The probability of fetal death increases		6
to Know the diseases and abnormalities that child has if the pregnancy the beginning and if it turns out that the disease or deformation is serious it can be aborted		1,3	
Protection against future risks, and for the family comfort		1	
to know that fetus is healthy		1	

To know the proper treatment of the disease before birth and also for the treatment after birth if there is it is possible		1,2	
It is necessary to prepare the parents psychologically	some people may not trust this type of diagnosis	4	7
To prepare for potential health problems		2	
Prevention or control of some genetic diseases.		1	
Get rid of pregnancy		3	
treat what can be treated or take the abortion decision		1,3	
treatment if possible or abortion		1,3	
Reducing the birth of children with incurable diseases		1	
Find out if there is a risk to the pregnant mother or fetus and take the necessary intervention if it is possible		1	
Checking the Safety of the fetus		1	
Find the appropriate treatment before birth if possible		1	
Knowing the disease and taking the necessary steps	legally and morally issues	1,2	8
Avoiding diseases		1	
The possibility of treating the fetus as soon as possible...	The disadvantages of the fetus for examination and abortion	1	6
Having an idea	Frustration	4	5
Early intervention		1	
	Mother 's anxiety in the case of an affected child and error during the diagnosis		5

To know what is the disease, its causes and how to avoid it in the future, treat what is possible, increase the awareness about such diseases and avoid them before they occur		1,2	
The possibility of treatment		1	
preparation of the parents for the child	they might not accept the child	4	5
The opportunity of abortion in the case of very severe disability - educating the parents about the child's disease and how to deal with it after birth		2,3	
Knowing of diseases and try to treat them early	and negatives are inaccuracy diagnosis	1	7
Avoiding the genetic diseases and get a strong generations		1	
It’s provide early checkup and enable detection of any serious disorder. Allowing the parent to decide wither to continue or go for abortion.		1,3	
Intervention for the treatment	panic of the parents	1	5
Psychological readiness and educate the parents about the disease and the ways to deal with it and the special needs that that be available		2,4	
To know the type of disease		1	
Disease prevention		1	
According to its accuracy, if it is very accurate in the diagnosis, it will help in early treatment		1	
The birth of a child in a specialist hospital therefore receives the necessary treatment.	stress of the mother	2	5
Avoid diseases and prevent them		1	
	not accurate		7

Knowing what diseases can occur before fetal development and there might be a treatments or steps to prevent it from happening		1	
Preparing of the parents		4	
Knowing the diseases before the birth and control or treat them		1,2	
The possibility of aborting the child in the absence of the necessary facilities for his care		3	
Giving you the chance to choose whether to keep the fetus or to abort it		3	
If the disease is cleared from the beginning of pregnancy, abortion is better than suffering		3	
	The possibility of aborting the fetus if it has deformities		10
Disease prevention		1	
Reassuring fetus health status		1	
Avoiding to have affected children		1	
knowing the genetic diseases that you have		1	
To avoid any diseases or malformations early before the completion of pregnancy		1	
Full knowledge of the baby health status		1	
parents preparation		2,4	
Knowing the diseases		1	
Helps to know the gender of the fetus and if there are any disabilities so the mother can decide is she wants to complete the pregnancy or abortion	Inaccurate test	1,3	7

Preparing parents before the birth of the fetus - Educating the parents about the state of health - Taking appropriate medical procedures for the situation		2	
Avoid the birth of a disabled child		1	
Avoiding deformities and diseases		1	
Keeps the baby safe		1	
prenatal diagnosis can prevent any problem that may occur		1	
Disease detection and treatment		1	
prevent the problem from the beginning	the mother will be stressed during pregnancy	1	5
Checking the health of the fetus		1	
Parents psychologically prepared and take commitments		2,4	
Know the problem before it happens and prevent it, because mother and child both will suffer if the child has a genetic disease		1,3	
Find out if the fetus is affected or not		1	
Avoiding and reducing the triggers of the disease		1	
It is preferable to prevent some diseases		1	
Psychological readiness		4	
Take precautions during the pregnancy		1	
Avoiding fetal disease and keeping the mother safe		1	
Reduction of fetal deformities		1	

preparing for the affected fetus	worsen the psychological state of the parents	4	5
checking the health status of the fetus	Abortion is one of the most common solutions	1	10
Knowing the child's health status and predict if there is a disability		1	
Find treatments and solutions if possible		1	
Either by abortion if parents are not ready, or they can prepare for it.	There may be a lack of respect for each other's feelings, sometimes a psychological deterioration of the parents	3,4	5
Having a healthy child		1	
Knowing the situation		1	
Ensure the fetus is free of genetic diseases and opportunity for treatment in the early stages	Parents worried about the idea of having a baby	1	5
helps to identify and treat genetic diseases before the birth		1	
Prevention of having a child with a genetic disease		1	
Gives the opportunity to the parents to be prepared and take the right decision		2,4	
very important, and if my mother did it will not be sick right now		1	
it will relieve much pain from my child		1	
To determine the type of disease		1	
Avoid the birth of children with disabilities		1	

The mother will be psychologically ; and also there is a chance for the mother to abort the fetus early if there is no harm to her health	I refused to do the prenatal diagnosis of the genetic diseases in my fourth month of pregnancy because they wanted take a sample from the fluids surrounding the fetus and I was worried about my baby	3,4	6
To discover the disease early		1	
	Unfortunately there is no explicit information about the effect of prenatal diagnosis		6.7
In order to avoid diseases		1	
To know what is going to happen and take the appropriate decision		2	
Decrease the incidence of genetic diseases		1	
Educate family about the disease And how to deal with it.	may cause abortion	2	8
Avoiding the genetic disease		1	
Early knowing of the condition and prepare for it		1,2	
Early diagnosis of genetic diseases		1	
Knowing the abnormality during the pregnancy		1	
early intervention of genetic diseases		1	

Decrease the incidence of having affected child and saving money and effort associated with it	Psychological pressure because of losing the baby	1	5
	If abnormality is confirmed the mother might takes decision of abortion		10
Knowing the health status of the fetus and and get prepared for it		1,2	
It might help to do medical intervention and prevent the abnormality		1	
Familiarity about the fetus status		1	
	Stressful		5
knowing how to deal with the abnormality in the future		2	
Familiarity with the fetus health status		1	
It can prevent some diseases		1	
Having fully diagnosis of the fetus health status	The feelings and the stress when the family know	1	5
opportunity for abortion	The prenatal diagnosis might be not accurate	3	7
Avoiding having affected children		1	
treatment the diseases before it gets worst	parents will be unhappy if there is no treatment for their child	1	5

Prevent or treat the abnormalities in this stage of life		1	
Follow up the fetus status to prevent diseases		1	
Provide opportunity for abortion		1,3	
Decrease the consequences of the disease and prepare the family		1,2	
Diagnosis of the genetic diseases		1	
Psychosocial preparation for the affected child		2,4	
Diagnosis of fetus abnormalities		1	
Early diagnosis	it might be harmful to the fetus	1	6
To get the parents psychologically prepared and prevent any preventable complication		1,4	
	It might be harmful to the pregnant woman		6
	It makes us afraid of the challenges and difficulties t		5
It might help to avoid some abnormalities		1	
To know the condition and decide what to do		1,2	
Psychological readiness	Psychological pressure	2,4	5
Provide early diagnosis and allow fast intervention and decision	Some prenatal diagnosis	1,2	6

making	procedures are invasive		
	Difficult to take a decision		5
	It might affect the pregnancy		6
Early prediction of disease	Not sure if the diagnosis is completely true or not	1	7
Give the mother choice to keep the baby or make the abortion	The intervention can cause fetal death	3	6
Get familiar with the situation and get prepared before birth		1,2	
It can prevent some diseases		1	
Parents gets prepared for the affected child		2,4	
To reassure the fetus		1	
It allows doctors to be informed about the babies situation	Some ways of diagnosis may cause several injuries to the baby	1	6
Mother readiness for the sick child		4	
Mother readiness for the sick child		4	
To know the fetal health status		1	
	Associated anxiety		5
Getting ready and prepared for the disease	It might be harmful to the fetus	2,4	6
Knowing the diseases that might affect my child	It might be harmful to the fetus	1	6
The mother can decided either accepting the situation or taking other actions	Makes the mother anxious and afraid of the future	2,3	5
